# Multiple cobalt active sites evenly embedded in mesoporous carbon nanospheres derived from a polymer-metal-organic framework: efficient removal and photodegradation of malachite green†

**DOI:** 10.1039/d2ra04906f

**Published:** 2022-11-10

**Authors:** Shuai Zhang, Hao Dang, Feilong Rong, Shunjiang Huang, Minghua Wang, Lijun Hu, Zhihong Zhang

**Affiliations:** College of Material and Chemical Engineering, Zhengzhou University of Light Industry Zhengzhou 450001 China 2006025@zzuli.edu.cn

## Abstract

A series of robust photocatalysts of mesoporous carbon nanospheres embedded with multiple cobalt active sites (Co/Co_*x*_O_*y*_@mC) have been constructed for efficient removal and photodegradation of malachite green (MG). Here, a cobalt-based polymeric-metal–organic framework (polyMOF(Co)) was constructed by using a polyether ligand containing 1,4-benzenedicarboxylic acid units. Afterward, polyMOF(Co) was calcined into a series of Co/Co_*x*_O_*y*_@mC hybrids at diverse high temperatures (400, 600, and 800 °C) under a N_2_ atmosphere. Therefore, Co coordination centers were transformed into various active sites such as Co, CoO, and Co_3_O_4_, which were embedded within the mesoporous carbon network derived from the polymeric skeleton. Considering the even distribution of Co-related active species and high porosity inherited from polyMOF(Co), the constructed Co/Co_*x*_O_*y*_@mC hybrid obtained at 600 °C illustrated higher removal ability (79%) with a maximum adsorption capacity of 314 mg g^−1^ within 120 min and better photodegradation performance (degradation rate of 95%) toward MG than those of the other photocatalysts obtained at 400 and 800 °C. Moreover, the possible photocatalytic reaction mechanisms, including the transfer behavior of charge carriers, generation of reactive species, and intermediate degradation of products, were provided. The present work showed an alternative strategy for the feasible and efficient preparation of photocatalysts based on MOFs.

## Abstract 2

The derivative catalysts of polyMOF(Co) effectively degrade malachite green under visible irradiation.

## Graphical abstract

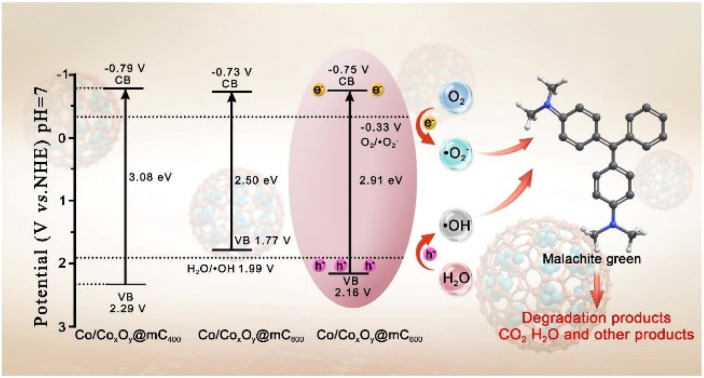

## Introduction

Malachite green (MG), a common synthetic triphenylmethane organic dye, is extensively employed for dyeing textiles and treating microbial infections.^1^ High-level MG greatly reduces the utilization of sunlight in water, weakens the photosynthetic efficiency of aquatic plants, and decreases oxygen content in water; thus, the removal and the degradation of MG are necessary.^2^ To date, various processes such as adsorption,^3^ photocatalysis,^4^ Fenton catalysis,^5^ and ozonation^6^ have been developed for treating MG. Amongst these processes, adsorption using efficient adsorbents is often utilized for the dislodge of organic dyes from wastewater systems.^7^ In addition, the adsorbed dye molecules can be recycled because of their high commercial value. As an effective, sustainable, and environmental-friendly technique, photocatalytic degradation is often applied for the elimination of MG from aqueous solution by coupling with adsorption.^8^ However, the big challenge is the development of efficient catalysts, which simultaneously possess adsorption and catalytic abilities toward organic dyes. At present, different photocatalysts such as rGO/CuS,^9^ chitosan/ZnO and chitosan/Ce–ZnO composites,^10^ nZVI/BC, melon/TiO_2_,^11^ diatomite/NiO composite,^12^ and multicopper BSA-Cu complex^13^ have been developed and used for efficiently removing and photodegrading MG. However, these nanomaterials or nanocomposites, which were prepared using the fussy preparation method, only demonstrate the sole ability, for example, adsorption or catalytic performance. Therefore, bifunctional catalysts with high adsorption capacities and photodegradation efficiency for treating MG under visible light must be further explored.

Recently, metal–organic frameworks (MOFs) have shown potential and extensive applications as efficient photocatalysts for adsorbing or degrading pollutants, such as Ti-MOF,^14^ Cu-MOF,^15^ and Co-MOF,^16^ because of their large surface area, tailorable chemistry, and rich functionality.^17^ MOF-based catalysts have also exhibited good catalytic ability toward the degradation of organic dyes. For example, the magnetic composite Fe_3_O_4_@MOF_UiO66_@TzDa-COF exhibited an efficient and rapid photocatalytic degradation effect of MG and Congo Red dyes.^18^ Furthermore, MIL-101(Fe) has been used as an efficient photocatalyst for activating peroxymonosulfate to remove organic dyes from aqueous solution.^19^ In addition, a three-dimensional Cu-MOF with a mesoporous structure has demonstrated dual functionalities in the removal and degradation of organic dyes.^20^ However, only few MOF-based photocatalysts demonstrate good degradation and absorption abilities of MG. Considerable attentions should be focused on the development of MOF-based nanomaterials to achieve the efficient degradation of MG under a complex environment.

The photodegradation efficiencies of most pristine MOF-based photocatalysts are unsatisfactory because of relatively short lifetime of photo-generated carriers and low photo absorption ability/charge utilization. Thus, diverse strategies have been used for preparing MOF-based photocatalysts such as bimetallic MOFs,^21^ MOF@MOF hybrids,^22^ MOF@COF hybrids,^23^ and MOF-based composites,^24^ which display improved photocatalytic performances. Recently, MOFs have been extensively utilized as precursors for the preparation of diverse metal catalysts in catalytic fields.^25^ MOF-based derivatives not only show initial morphologies of their parents, but also more exposed active sites and larger surface area^26^ than pristine MOFs. In generally, various nanocomposites or hybrids of metal compounds (phosphides, sulfides, or selenides) and mesoporous carbon networks can be obtained during calcination of MOFs at high-temperature under inert atmosphere.^27^ Compared with MOFs or MOFs-based composites, MOFs-derived composites exhibit enhanced degradation performance toward pollutants because of the good catalytic ability of these transitional metal compounds.^28^ On the contrary, mesoporous carbon frameworks can also boost electron transfer, thereby shortening electron transport path.^29^ Given the large specific surface area, mesoporous structure, abundant active sites, and superior photo-electron transfer, the construction of MOF-based derivatives could efficiently degrade or adsorb MG. However, annealing MOFs at high temperature often leads to the serious accumulation of metal nanoparticles (NPs), thereby decreasing their dispersion in aqueous solution and their degradation ability toward organic dyes.^30^ In addressing this issue, many sacrificial substances such as organic molecules, graphene, carbon nitrides, and polymers have been utilized as the scaffolds for metal NPs derived from MOFs.^31^ Although the formed additional carbon networks can avoid the accumulation of metal NPs to improve catalytic performances, such networks would remarkably aggravate the difficulty in the analysis of chemical structures and components of these nanomaterials obtained by this preparation strategy *via* the calcination of the mixtures of MOFs and other components. Therefore, seeking novel MOF-based precursors that contain rich organic components is important for the manufacture of ascendant photocatalysts for efficient degradation and removal of organic dyes.

A crystalline polymeric MOF was synthesized using an amorphous polymer (denoted as polyMOF),^32^ in which the MOF regularity can reserved by using alkyl chain spacers. Compared with MOFs synthesized by using small organic molecules as ligands, the integration of a long polymer chain and MOFs of polyMOFs can result in a category of processable MOFs. Moreover, long polymer chains containing polyMOFs can serve as carbon source when polyMOFs are calcined at high temperature, which would be used as a robust scaffold for the even distribution of formed metal NPs. Furthermore, cobalt oxides (CoO_*x*_) exhibit the p-type semiconducting nature, and they can serve as hole collectors and photoactive sites.^33^ Thereto, various CoO_*x*_-relevant composites have been fabricated and utilized as photocatalysts.^34^ Based on these encouraging results, a novel polyMOF(Co) network was synthesized using the polyether ligand containing 1,4-benzenedicarboxylic acid (H_2_BDC) units as part of the polymer backbone as building block. The obtained polyMOF(Co) was composed of spheres with different sizes, and it possessed large specific surface area, large pore size, mixed valence states of cobalt (Co^2+^/Co^3+^), and rich functional moieties (CH_*x*_, COO, C=O, or C–O). By calcining at various high temperature under N_2_ atmosphere (400 °C, 600 °C, and 800 °C), the obtained polyMOF(Co) spheres were transformed into diverse active metal NPs or nanocrystals (such as Co, CoO, and Co_3_O_4_) homogeneously embedded in the mesoporous carbon scaffold (referred as Co/Co_*x*_O_*y*_@mC), maintaining the spherical shape. Given that Co/Co_*x*_O_*y*_ NPs exhibited superior catalytic ability toward pollutants^35,36^ and the high adsorption performance of mesoporous carbon network,^37^ the gained Co/Co_*x*_O_*y*_@mC nanohybrid demonstrated the bifunctional capacities for simultaneously photodegrading and adsorbing MG from polluted water. Based on previous reports, various cobalt-based catalysts have been achieved from MOFs *via* the calcination method and exhibited the outperformed treated abilities for various pollutants.^38,39^ Nonetheless, few works focused on the application of polyMOFs in the environment restoration field. By contrast, the Co/Co_*x*_O_*y*_@mC_600_ hybrid obtained by annealing polyMOF(Co) at 600 °C illustrated superior adsorption ability and catalytic performances to other nanohybrids. In addition, varieties analysis techniques have been explored to reveal the formation mechanism of the obtained Co/Co_*x*_O_*y*_@mC, including the distribution of active catalytic sites, chemical components, and crystal and nanostructure. Moreover, the adsorption and photodegradation mechanisms and the degradation products of MG were investigated by treating with the Co/Co_*x*_O_*y*_@mC_600_ catalyst. The present work can provide insights into the construction of effective bifunctional photocatalysts for absorbing and degrading pollutants.

## Results and discussion

### Basic characterizations of the series of samples

**Fig. 1:**
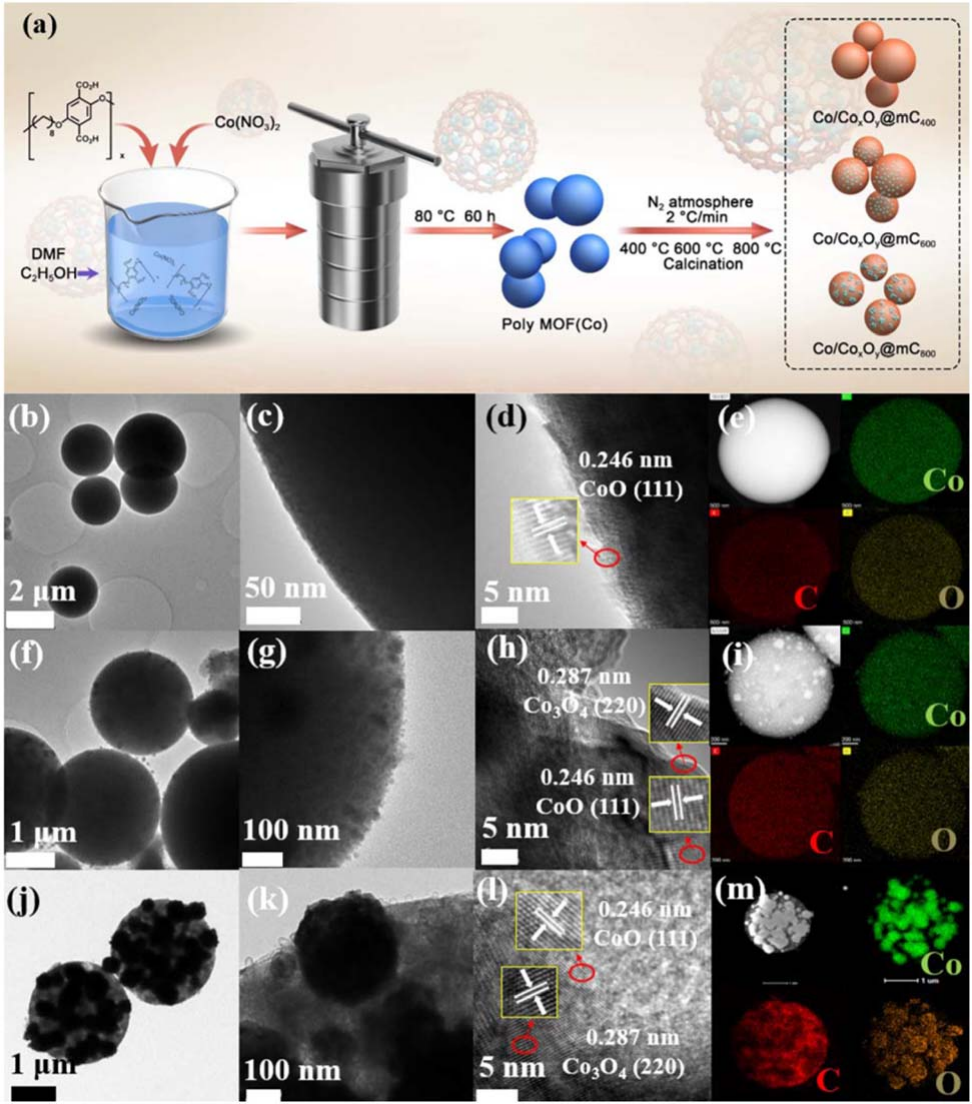
(a) Schematic illustration of the preparation of polyMOF(Co) and the series of Co/Co_*x*_O_*y*_@mC by calcining polyMOF(Co) at different temperature of 400 °C, 600 °C, and 800 °C under N_2_ atmosphere. TEM images and HAADF image, element mappings (Co (green), C (red), and O (orange)) of (b–e) Co/Co_*x*_O_*y*_@mC_400_, (f–i) Co/Co_*x*_O_*y*_@mC_600_, and (j–m) Co/Co_*x*_O_*y*_@mC_800_ hybrids.

The basic characterizations of polyMOF(Co) and Co/Co_*x*_O_*y*_@mC hybrids were performed to investigate the surface morphologies and nanostructures. The S2 section (ESI†) shows that polyMOF(Co) exhibits a regular spherical structure, but with diverse sizes of 1–2 μm (Fig. S1†). As shown in Fig. S3a and b,† Co/Co_*x*_O_*y*_@mC_400_ shows a smooth spherical structure of its parent, polyMOF(Co), which can be proved by its TEM image (Fig. 1b and c). Nonetheless, some small particles embedded within the interior of spheres can be found indistinctly. The high-resolution-TEM (HR-TEM) image clearly shows the lattice spring of 0.246 nm, corresponding to CoO.^40^ This finding suggests that part of the outer layer of polyMOF(Co) was oxidized to metal oxide at 400 °C. As for Co/Co_*x*_O_*y*_@mC_600_, large amounts of spheres can be reserved, but it has a rougher surface than polyMOF(Co). Abundant small particles are embedded in Co/Co_*x*_O_*y*_@mC_600_ spheres, indicating spheres were decomposed when pyrolyzing at high temperature (600 °C) (Fig. S3c and d†). The TEM image of Co/Co_*x*_O_*y*_@mC_600_ distinctly displays intact spheres, with a few of small NPs on the surface and interior of spheres (Fig. 1f). Evident lattice springs of 0.246 and 0.287 nm are observed in the HR-TEM image of Co/Co_*x*_O_*y*_@mC_600_ (Fig. 1h), corresponding to CoO and Co_3_O_4_, respectively.^41^ Co nodes in the polyMOF(Co) network were oxidized to Co_*x*_O_*y*_ when calcined at high temperature under N_2_ atmosphere. Moreover, part of polymer ligands were decomposed and they formed CO, CO_2_, or other small gas molecules, which escaped from the spheres, thereby leading to the porous structure of the gained hybrid, whereas the reserved polymer ligands were transferred into graphitized carbon. Notably, the generated graphitized carbon can remarkably promote the electron transport and hamper the agglomeration of metal NPs. This occurrence becomes evident when annealing polyMOF(Co) at 800 °C. In this case, large NPs were aggregated in Co/Co_*x*_O_*y*_@mC_800_ spheres, whereas some particles were leaked from spheres, reserving the holes within the whole spheres (Fig. S3e and f†).

Furthermore, the TEM image of the Co/Co_*x*_O_*y*_@mC_800_ hybrid shows that some large NPs are embedded within the spheres, whereas the color of the other part becomes light, indicating the porous structure (Fig. 1j). In addition, some nanotubes can be found in Co/Co_*x*_O_*y*_@mC_800_, along with large amount of particles (Fig. 1k). The HR-TEM image (Fig. 1l) shows lattice springs of 0.246 and 0.287 nm, which correspond to CoO and Co_3_O_4_, respectively. Moreover, STEM-energy dispersive X-ray (STEM-EDX) elemental mapping and EDX spectrum of all Co/Co_*x*_O_*y*_@mC hybrids were investigated (Fig. 1 and S3†). The results reveal that Co, C and O elements are homogeneously dispersed in the whole selected region of all hybrids. The EDX spectra (Fig. S3c, f, i and Table S1†) demonstrate that the C content in Co/Co_*x*_O_*y*_@mC_400_, Co/Co_*x*_O_*y*_@mC_600_, and Co/Co_*x*_O_*y*_@mC_800_ increases, that is, 57.24%, 65.37% and 69.99%, respectively. Moreover, the atomic% of O in a series of Co/Co_*x*_O_*y*_@mC hybrids decreases from 21.81% to 16.48% along with the increase of calcination temperature. Further, the inductively coupled plasma mass spectrometry (ICP-MS) was employed to determine the atomic% of Co content in Co/Co_*x*_O_*y*_@mC_400_, Co/Co_*x*_O_*y*_@mC_600_, and Co/Co_*x*_O_*y*_@mC_800_. As listed in Table S1,† the Co content of Co/Co_*x*_O_*y*_@mC_400_ is around 15.5%, higher than that of polyMOF(Co) due to the evaporation of residue of water and solvent. The Co content in Co/Co_*x*_O_*y*_@mC_600_, and Co/Co_*x*_O_*y*_@mC_800_ become higher, approximately 18.11% and 18.43%, respectively, due to the degradation of polymer ligand.

**Fig. 2:**
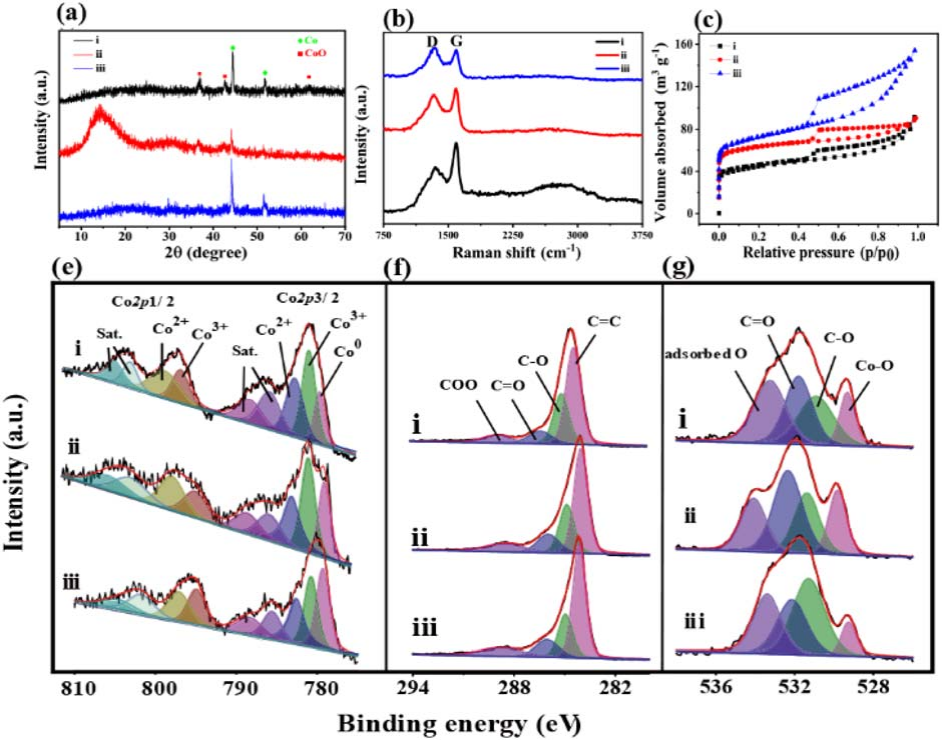
(a) XRD patterns, (b) Raman spectra, (c) N_2_ adsorption–desorption isotherms, and the high-resolution (e) Co 2p, (f) C 1s and (g) O 1s XPS spectra of (i) Co/Co_*x*_O_*y*_@mC_400_, (ii) Co/Co_*x*_O_*y*_@mC_600_, and (iii) Co/Co_*x*_O_*y*_@mC_800_.

The XRD pattern of Co/Co_*x*_O_*y*_@mC_400_ (curve i, Fig. 2a) displays two peaks at 2*θ* = 44.2° and 51.6°, indicating the (111) and (200) facet of metallic Co (JCPDS No. 15-0806), respectively. In addition, three additional peaks at the 2*θ* = 36.6°, 42.4° and 61.5° are found because of the (111), (200) and (220) crystal planes of CoO (JCPDS No. 48-1719), respectively. These results indicate that the partial cobalt nodes in polyMOF(Co) were reduced to metallic Co, whereas the other partial cobalt sites were oxidized into metal oxide state (CoO) when calcined at 400 °C, which are consistent with the TEM results. However, when annealing at 600 °C, the peak intensities of Co and metal oxide decrease, with a wide clear peak at 2*θ* = 15.2° caused by graphite carbon.

This finding suggests that the organic network of polyMOF(Co) was further decomposed and carbonized. The formed mesoporous carbon spheres not only can enhance electron transfer, but also promote photocatalytic degradation and adsorption ability of the hybrid.^42^ The XRD pattern of Co/Co_*x*_O_*y*_@mC_800_ also includes the diffraction peaks of Co and CoO but without the wide peak of graphite carbon. As aforementioned, most of carbon components of polyMOF(Co) were decomposed and transformed into graphite carbon by annealing at high temperature. At extremely high temperature, the carbon component was further dissociated into small molecules (CO_2_ or CO), thereby decreasing the content of graphite carbon.

Moreover, Raman spectra of the series of Co/Co_*x*_O_*y*_@mC hybrids exhibit two substantial peaks located at 1342 and 1596 cm^−1^ (Fig. 2b), which are assigned with D- and G-bands of graphitic carbon, respectively. The D band represents the defect of C atom lattice, while the G-band indicates the in-plane stretching vibration of C atom sp^2^ hybrid. The intensity ratios of the two peaks of D- and G-bands, *I*_D_/*I*_G_, represent the degree of defect of graphitic carbon. The *I*_D_/*I*_G_ value of a series of Co/Co_*x*_O_*y*_@mC hybrids decreases with increase of carbonization temperature, that is, 1.01, 0.96, and 0.78, respectively. As aforementioned, the degree of defect of graphitic carbon in Co/Co_*x*_O_*y*_@mC_800_ is the lowest among the three samples. Furthermore, the Co/Co_*x*_O_*y*_@mC_800_ hybrid exhibits a broad peak at the center of 2795 cm^−1^, which is ascribed to 2D band. Notably, the 2D-band is a typical symbol of ordered graphitic carbon,^43^ indicating the highly ordered carbon in Co/Co_*x*_O_*y*_@mC_800_.

The Brunauer–Emmett–Teller (BET) nitrogen adsorption–desorption measurements were performed at 77 K to characterize the specific surface areas and pore structures of Co/Co_*x*_O_*y*_@mC hybrids (Fig. 2c and d). All catalysts display the typical IV-type isotherm with H_3_ hysteresis loops, indicating their mesoporous structure. Evidently, Co/Co_*x*_O_*y*_@mC_800_ has a large specific surface area (264 m^2^ g^−1^), which is close to that of Co/Co_*x*_O_*y*_@mC_600_ (236 m^2^ g^−1^) and larger than that of Co/Co_*x*_O_*y*_@mC_400_ (161 m^2^ g^−1^) (Table S2†). By contrast, the specific surface area of these hybrids is larger than that of polyMOF(Co) (85.7 m^2^ g^−1^). Among the three samples, the average pore size of Co/Co_*x*_O_*y*_@mC_600_ is the smallest (2.37 nm) (Fig. S4†). The larger specific surface area with an abundant mesoporous structure of Co/Co_*x*_O_*y*_@mC can provide more active sites and facilitate adsorption/desorption of reactants and products on the surface of catalysts, providing excellent adsorption and photocatalytic performance.

The chemical structures and components of pristine polyMOF(Co) spheres and a series of Co/Co_*x*_O_*y*_@mC hybrids were also investigated by XPS. The XPS survey scan spectra of all samples (Fig. S5†) include the signals of Co 2p (780.3 eV), C 1s (285 eV) and O 1s (531 eV). The high-resolution XPS of each element of all samples, that is, Co 2p, C 1s, and O 1s, were analyzed by Gaussian fitting (Fig. 2 and S2†). The Co 2p XPS spectrum of the pristine polyMOF(Co) (Fig. S2f†) can be split into two couples of peaks at the binding energy (BE) centers of 783.8 and 800 eV, corresponding to Co 2p_3/2_ and Co 2p_1/2_, respectively. The Co 2p_3/2_ part is composed of the peaks of 780.9 and 782.4 eV, corresponding to Co^3+^ and Co^2+^ species, respectively, and their shakeup satellite peaks at BEs of 785.2 and 787.6 eV, respectively. The analogous components are found in the Co 2p_1/2_ part, showing the peaks at the BEs of 796.6, 797.9, 801.4, and 803.9 eV, which are assigned to Co^3+^, Co^2+^, and their satellite peaks. This finding indicates that the mixed Co^2+^ and Co^3+^ valence states are present in polyMOF(Co). By contrast, apart from Co^3+^ and Co^2+^ species, the resembled deconvoluted peaks of the high-resolution Co 2p XPS spectra of Co/Co_*x*_O_*y*_@mC_400_, Co/Co_*x*_O_*y*_@mC_600_, and Co/Co_*x*_O_*y*_@mC_800_ exhibit one additional peak at the BE of 778.9 eV, which is due to the metal state of Co (Co^0^) (Fig. 2e). This finding indicates that part of the Co species in polyMOF(Co) were reduced to metallic Co^0^. Based on the ratio of the peak area of Co species to the sum peak area of the Co 2p_3/2_ part, the content of Co^0^ species in Co/Co_*x*_O_*y*_@mC_400_, Co/Co_*x*_O_*y*_@mC_600_, and Co/Co_*x*_O_*y*_@mC_800_ is approximately 13.4%, 19.9%, 27.5%, respectively. The relatively large content of Co^0^ can greatly facilitate electron transfer and improve the catalytic ability.^44^ Moreover, based on the peak areas of Co^3+^ and Co^2+^ species in the Co 2p_3/2_ species, the ratios of Co^3+^/Co^2+^ in Co/Co_*x*_O_*y*_@mC_400_, Co/Co_*x*_O_*y*_@mC_600_, and Co/Co_*x*_O_*y*_@mC_800_ are 1.22, 1.24, 1.06, respectively. The higher Co^3+^/Co^2+^ ratio in interfacial Co/Co_*x*_O_*y*_@mC is favorable for the generation of active radical to promote degradation process.^45^ Furthermore, these Co/Co_*x*_O_*y*_@mC hybrids comprise Co^3+^, Co^2+^ and Co^0^ species, which are conductive to electron transfer and enhanced catalytic ability.^46^ In addition, the C 1s XPS spectrum of polyMOF(Co) is composed of five main components at the BEs of 284.0, 284.6, 285.2, 286.2, and 288.5 eV, corresponding to C=C, C–C, C–O, C=O, and COO, respectively (Fig. S2g†), which are originated from the polymer ligand in polyMOF. Moreover, a weak peak at the BE of 291.9 eV ascribing to the π–π* bond is observed, which is due to benzene ring bearing on polymer chain. The C 1s XPS spectra of the series of Co/Co_*x*_O_*y*_@mC hybrids can be separated into four parts of C=C (284.0 eV), C–O (285.2 eV), C=O (286.2 eV) and COO (288.5 eV) (Fig. 2f). The appearance of C=C group with a large peak area in a series of Co/Co_*x*_O_*y*_@mC hybrids further verifies the highly conjugated structure, which may be due to the graphitic carbon and may boost the electron transfer.^47^ By contrast, the declined peak areas of COO and C=O groups indicate that these carbon-related moieties were decomposed by calcining at high temperature. In addition, the O 1s XPS spectrum of polyMOF(Co) only includes three peaks at the BEs of 531.5, 532.5, and 533.3 eV (Fig. S2h†), corresponding to C=O, C–O and adsorbed O, respectively. Nonetheless, four deconvoluted peaks are obtained in the O 1s spectra of a series of Co/Co_*x*_O_*y*_@mC hybrids, including Co–O (529.3 eV), C–O (531.3 eV), C=O (532.14 eV), and adsorbed O (533.4 eV) (Fig. 2g). These results indicate that Co coordination sites in polyMOF(Co) were oxidized to metal oxide during the calcination. By contrast, the content of Co–O in the Co/Co_*x*_O_*y*_@mC_600_ is the highest among the three hybrids, approximately 17.5% of oxide-contained groups, which is substantially lower than those of Co/Co_*x*_O_*y*_@mC_400_ (8.1%) and Co/Co_*x*_O_*y*_@mC_800_ (13.5%). As aforementioned, the Co content in Co/Co_*x*_O_*y*_@mC_600_ is composed of Co_*x*_O_*y*_ and abundant metallic Co, which can remarkably enhance the photodegradation ability of catalyst.

### Photo-electron performances of polyMOF(Co) and the series of Co/Co_*x*_O_*y*_@mC hybrids

**Fig. 3:**
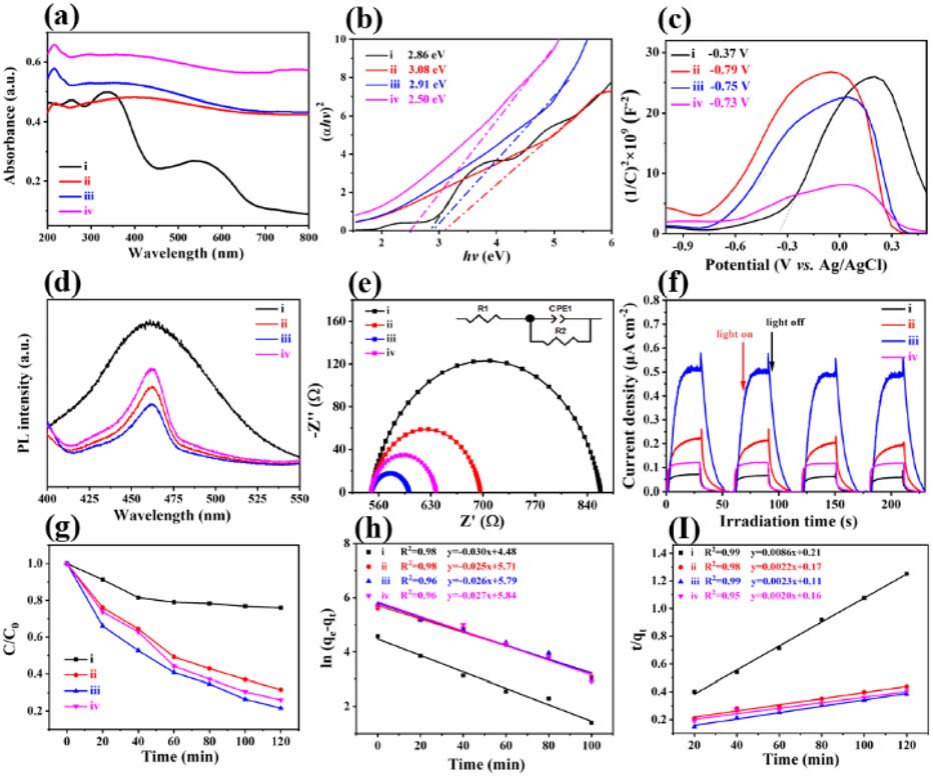
(a) UV-vis diffuse reflectance spectroscopy, (b) the plots of (*αhν*)^2^*versus* photo energy (*hν*), (c) Mott–Schottky plots, (d) PL spectra, (e) Nyquist plots of electrochemical impedance spectroscopy (inset: equivalent circuit model), (f) photocurrent response curves, (g) adsorption rate of MG on different adsorbents, (h) pseudo first order adsorption kinetic curves of different adsorbents, and (i) pseudo second order adsorption kinetic curves of different adsorbents of (i) polyMOF(Co), (ii) Co/Co_*x*_O_*y*_@mC_400_, (iii) Co/Co_*x*_O_*y*_@mC_600_, and (iv) Co/Co_*x*_O_*y*_@mC_800_.

The optical absorption properties of polyMOF(Co) and a series of Co/Co_*x*_O_*y*_@mC hybrids were investigated by UV-vis diffuse reflectance spectroscopy (DRS) (Fig. 3a). Based on the Kubelka–Munk function, the band gap energies (*E*_g_) of polyMOF(Co), Co/Co_*x*_O_*y*_@mC_400_, Co/Co_*x*_O_*y*_@mC_600_, and Co/Co_*x*_O_*y*_@mC_800_ catalysts can be deduced in accordance with the plot of (*αhν*)^2^*versus hν* (Fig. 3b), which are 2.86, 3.08, 2.91, and 2.50 eV, respectively. Furthermore, Fig. 3c shows the Mott–Schottky curves of the constructed catalysts. Their positive slopes indicate that these catalysts are n-type semiconductors. Based on the flat band potentials, the conduction band (CB) positions can be estimated according to the *X*-intercepts of the linear regions of their Mott–Schottky curves.^48^ Consequently, the flat band positions of polyMOF(Co), Co/Co_*x*_O_*y*_@mC_400_, Co/Co_*x*_O_*y*_@mC_600_, and Co/Co_*x*_O_*y*_@mC_800_ samples are −0.37, −0.79, −0.75 and −0.73 V *vs.* Ag/AgCl, respectively, that is, −0.17, −0.59, −0.55 and −0.53 V (*vs.* NHE).

Notably, the CB potential (*E*_CB_) for n-type semiconductor has a more negative value (0.1–0.2 V) compared with the flat band. Thus, the CB bottom potentials of polyMOF(Co), Co/Co_*x*_O_*y*_@mC_400_, Co/Co_*x*_O_*y*_@mC_600_, and Co/Co_*x*_O_*y*_@mC_800_ catalysts are estimated to be −0.37, −0.79, −0.75 and −0.73 V, respectively. Given the band gaps of polyMOF(Co) (2.86 eV), Co/Co_*x*_O_*y*_@mC_400_ (3.08 eV), Co/Co_*x*_O_*y*_@mC_600_ (2.91 eV), and Co/Co_*x*_O_*y*_@mC_800_ (2.50 eV) calculated according to eqn (1)

1 *E*_VB_ = *E*_CB_ + *E*_g_,

their VB positions are calculated to be 2.49, 2.29, 2.16 and 1.77 eV (*vs.* NHE), respectively. Fig. 3d shows that polyMOF(Co) and a series of Co/Co_*x*_O_*y*_@mC hybrids exhibit a strong characteristic emission peak located at 463 nm, which is related to the recombination of the photo-induced carriers.^49^ By contrast, the peak intensity of Co/Co_*x*_O_*y*_@mC_600_ is the lowest, indicating that the electron hole recombination ability is the weakest, which is conductive to the improvement of the photocatalytic degradation ability of pollutants.

The electrochemical performance of one photocatalyst greatly reflects the electron transfer ability.^50^ Here, electrochemical impedance spectroscopy was used for the investigation of the electrochemical activities of all samples (Fig. 3e). Based on the equivalent circuit, the obtained resistance of charge transfer (*R*_ct_) of the Co/Co_*x*_O_*y*_@mC_600_ hybrid is the smallest (49.9 Ω) among all samples. This finding indicates the superior electrochemical performance, which can greatly facilitate electron transfer when carrying out the photocatalysis reaction. By contrast, polyMOF(Co) exhibits the largest *R*_ct_ of 308.4 Ω, indicating poor electrochemical activity. Therefore, the polymer ligand coordinated with Co ions substantially hamper electron transfer. When calcining at 400 °C, the conductivity of the hybrid is improved, resulting in a smaller *R*_ct_ (166.6 Ω) than that of polyMOF(Co), which is primarily ascribed to the partial transition of polymer ligands and Co ions. With regard to the Co/Co_*x*_O_*y*_@mC_800_ hybrid, the accumulation of Co and CoO inhibits electron transfer, resulting in a larger *R*_ct_ (83 Ω) than that of Co/Co_*x*_O_*y*_@mC_600_. Given the presence of graphite carbon in Co/Co_*x*_O_*y*_@mC_800_, its electrochemical activity is more outstanding than that of Co/Co_*x*_O_*y*_@mC_400_.

In studying the light-harvesting and separation efficiency of photo-generated electron–hole pairs, the photocurrent–time curves of all samples were performed with multiple 1 min light on–off cycles (Fig. 3f). The result shows that the photocurrent of the Co/Co_*x*_O_*y*_@mC_600_ hybrid is the highest, which is approximately 7.5, 2.3 and 4.1 times higher than those of polyMOF(Co), Co/Co_*x*_O_*y*_@mC_400_ and Co/Co_*x*_O_*y*_@mC_800_, respectively. The strong photocurrent intensity of the Co/Co_*x*_O_*y*_@mC_600_ hybrid indicates fast electron transfer.

### Adsorption abilities of polyMOF(Co) and series of the Co/Co_*x*_O_*y*_@mC hybrids

#### Adsorption dynamics of MG

The MG solution with initial concentration of 80 mg L^−1^ (50 mL) was used as the polluted solution for monitoring the adsorption capacity of diverse Co/Co_*x*_O_*y*_@mC hybrids by dispersing them in MG solution, following by stirring under dark condition. After taking 3 mL of the supernatant every 20 min, the catalyst was removed by centrifugation, for which the UV-vis adsorption at 426 nm was measured using a UV-vis spectrophotometer.

The removal rate was calculated as 
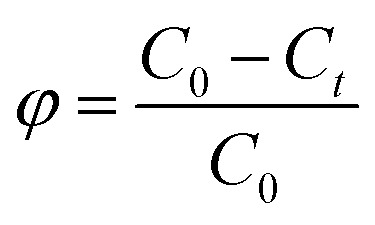
, and the maximum adsorption capacity was calculated as 
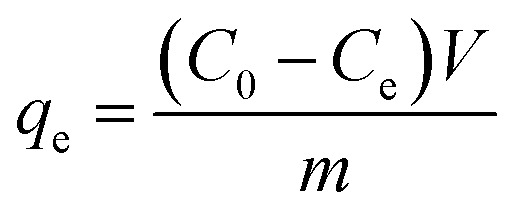
.

A series of Co/Co_*x*_O_*y*_@mC hybrids were used as adsorbents for the removal of MG from aqueous solution, whereas polyMOF(Co) was used for comparison. Fig. 3g indicates the percentage of MG removal of the Co/Co_*x*_O_*y*_@mC_600_ hybrid is 79% within 2 h, which is higher than those of Co/Co_*x*_O_*y*_@mC_800_ (75%), Co/Co_*x*_O_*y*_@mC_400_ (69%), and polyMOF(Co) (24%). Co/Co_*x*_O_*y*_@mC_600_ has a large specific surface area, which increases the contact with MG. In addition, the small pore diameter causes the MG to become stable. Furthermore, the comparison of the adsorption capacities (*q*_e_) of MG with different catalysts is summarized in Fig. S6,† in which the Co/Co_*x*_O_*y*_@mC_600_ catalyst illustrates the maximum adsorption capacity of 314 mg g^−1^, 3.27 times higher than that of polyMOF(Co). In addition, it is higher than that reported in the literature, including Lotus leaf (125.3 mg g^−1^),^51^ Spent-tea-leaves-based activated carbon (256.4 mg g^−1^),^52^ Palm-flower-based activated carbon (48.2 mg g^−1^),^53^ and Ricinus-communis-based activated carbon (27.8 mg g^−1^).^54^ The adsorption kinetic model is obtained using the following equations:^55^

2 Pseudo-first-order-kinetic-model: ln(*q*_e_ − *q*_*t*_) = ln *q*_e_ − *k*_1_*t*

3
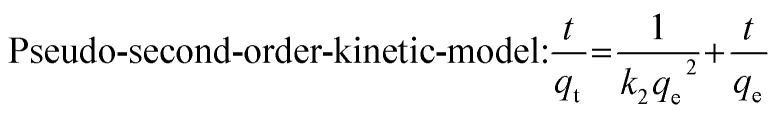
where *q*_e_ is the adsorption capacity at equilibrium; *q*_t_ is the adsorption capacity of each period; *k*_1_ is the first order dynamic constant; and *k*_2_ is the second order dynamic constant. The experimental results were fitted by using the pseudo first-order kinetic equation and pseudo second-order kinetic equation. All fitting results are shown in Fig. 3h and i and summarized in Table S3†. By contrast, the fitting parameter of pseudo second-order dynamics *R*^2^ is larger than that of the pseudo first-order dynamics *R*^2^, whereas the fitting parameters of the pseudo second-order dynamics *q*_e_ are closer to the test data. This result suggests that the adsorption behavior of the Co/Co_*x*_O_*y*_@mC catalysts for MG is consistent with the pseudo second-order kinetic equation. Therefore, the results indicate that the adsorption process of the Co/Co_*x*_O_*y*_@mC catalysts for MG is primarily controlled by chemical adsorption. This chemical adsorption includes the contribution of charge interaction and the influence of hydrogen bond.^56^

#### Influencing factors and adsorption models toward MG

**Fig. 4:**
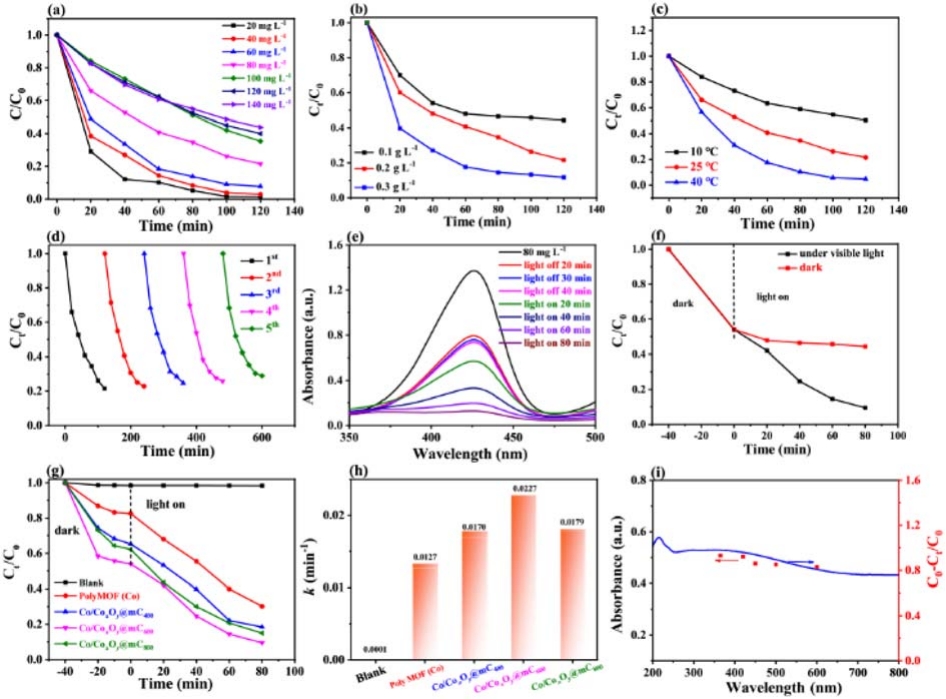
The effect of (a) different initial concentrations, (b) different amount of catalyst, (c) different system temperatures on malachite green adsorbent. (d) Repeatability for the MG. (e) UV-vis spectral absorption changes of MG solution photodegraded over Co/Co_*x*_O_*y*_@mC_600_ under visible light irradiation. (f) The comparison of Co/Co_*x*_O_*y*_@mC_600_ photocatalyst dark and light degradation of MG. (g) Photocatalytic degradation of MG. (h) The comparison of apparent rate constants of different photocatalysts, and (i) UV-vis DRS (blue line) and action spectrum (red plots) of MG degradation over Co/Co_*x*_O_*y*_@mC_600_ through different band pass filters. [MG] = 80 mg L^−1^, [Co/Co_*x*_O_*y*_@mC_600_] = 0.1 g L^−1^, [pH] = 7.0, [*T*] = 25 °C.

The optimal conditions for the MG adsorption using the Co/Co_*x*_O_*y*_@mC_600_ hybrid were investigated to obtain superior adsorption ability toward MG. Different experimental parameters such as the adsorbent dosage, system temperature, and initial concentration of MG were optimized. Fig. 4a shows the adsorption rate of MG with diverse initial concentrations. Notably, the adsorption rate of MG decreases with increasing of the initial concentration of MG. When the initial concentration is 20 mg L^−1^, almost all MG molecules are thoroughly adsorbed by Co/Co_*x*_O_*y*_@mC_600_, indicating saturated adsorption. When the concentration of MG solution gradually increases, the adsorption capacity also increases, which is close to the equilibrium value of 314 mg g^−1^. The effect of the dosage of the used adsorbent (*i.e.*, 0.1, 0.2, and 0.3 g L^−1^) on the adsorption performance was also probed (Fig. 4b). The result shows that the maximum adsorption ability of the adsorbent toward MG increases with increase of the adsorbent usage, in which Co/Co_*x*_O_*y*_@mC_600_ (15 mg mL^−1^) had the largest adsorption ability of 88% (352 mg g^−1^). This result is attributed to the abundant active sites of the large amount of adsorbent. With regard to the effect of the temperature on the adsorption efficiency (Fig. 4c), the adsorption capacity increases with increase of system temperature. The maximum adsorption ability of 95% was obtained when the system temperature is up to 40 °C.

Furthermore, the MG-polluted water system with different concentrations (381 mg g^−1^) was treated using the Co/Co_*x*_O_*y*_@mC_600_ hybrid (0.2 g L^−1^). Herein, the Langmuir equation and Freundlich equation^57^ were used to describe the adsorption isotherm of organic dyes.4
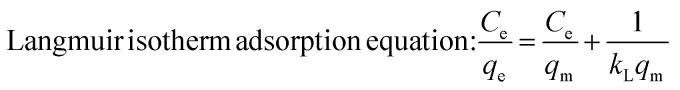
5
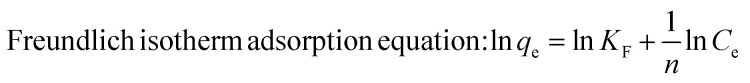
where *q*_e_ is the adsorption capacity at equilibrium, *q*_m_ is maximum monolayer adsorption capacity of adsorbent, *C*_e_ is the concentration of solution at equilibrium, *k*_L_ is Langmuir constant, *k*_F_ is isotherm constant for the characterization of adsorption capacity, and *n* is the isotherm constant characterizing strength.

All fitting results are shown in Fig. S7† and Table S2†. The *R*^2^ of Langmuir equation is approximate 0.9875, which is larger than that of Freundlich equation (0.8970). The adsorption behavior of MG on Co/Co_*x*_O_*y*_@mC_600_ was in accordance with the Langmuir isotherm model, indicating that the adsorption of MG is mainly attributed to the monolayer adsorption.^58^

For the practical application, the reusability of the used catalyst is an important indicator for evaluating the photocatalytic performance. After photocatalytic degradation, the catalyst was centrifuged and washed with ethanol three times and then dried for the next cycle. The repeated adsorption–desorption experiments were performed to evaluate the reusability of Co/Co_*x*_O_*y*_@mC_600_. Fig. 4d shows that the adsorption rate of MG reaches more than 80% of the original value, suggesting the excellent reusability of the developed adsorbent.

### Photodegradation performances of the series of Co/Co_*x*_O_*y*_@mC toward MG

#### Photodegradation activities of the series of Co/Co_*x*_O_*y*_@mC hybrids toward MG

Photocatalytic tests of a series of Co/Co_*x*_O_*y*_@mC hybrids were evaluated on the basis of the photodegradation of MG under visible light irradiation (*λ* > 420 nm). Fig. 4e shows the UV-vis absorption changes of the MG solution photodegraded by Co/Co_*x*_O_*y*_@mC_600_ under visible light irradiation. Notably, the peak intensity of the absorption peak at 426 nm decreases with the illumination time, indicating that MG molecules are gradually degraded. The MG degradation ability of the developed catalyst under dark condition was also probed (Fig. 4f), showing a removal rate of 55.8%, which is distinctly lower than that of the catalyst under visible-light irradiation. This finding indicates that the visible light plays an essential role in the catalytic ability of Co/Co_*x*_O_*y*_@mC_600_ toward MG. Fig. 4g shows the photodegradation efficiency of Co/Co_*x*_O_*y*_@mC_600_ is the highest (90.5%) among four samples toward MG (80 mg mL^−1^), whereas polyMOF(Co) only displays a low degradation rate of 69.8% under the same conditions. In addition, the evident rate constant (*k*) of MG degradation can be deduced using eqn (6):^59^

6 *k*·*t* = −ln(*C*_*t*_/*C*_0_)

As such, the *k* value of Co/Co_*x*_O_*y*_@mC_600_ is 0.0227 min^−1^, which is 1.78 times of polyMOF(Co) and apparently higher than those of Co/Co_*x*_O_*y*_@mC_400_ (0.0170 min^−1^) and Co/Co_*x*_O_*y*_@mC_800_ (0.0179 min^−1^) catalysts (Fig. S8†). Thus, Co/Co_*x*_O_*y*_@mC_600_ not only exhibits superior adsorption ability, but also demonstrates good photodegradation efficiency to polyMOF(Co). The apparent rate constants of different catalysts were compared to determine the effect of visible light on the catalytic performance, and the results are shown in Fig. 4h. Moreover, the removal efficiency of MG over Co/Co_*x*_O_*y*_@mC_600_ was also probed by separately irradiating with the light of diverse wavelength (light intensity 10 mW cm^−2^, Fig. 4i). The results show that the removal rate of MG follows the order of *λ* = 365 nm > *λ* = 420 nm > *λ* = 450 nm > *λ* = 500 nm > *λ* = 600 nm. Therefore, the short-wavelength light in the monochromatic light source can gradually enhance the photodegradation ability of MG caused by high energy in short-wavelength photons, which can result in the great decomposition of chemical bonds of MG molecules. Meanwhile, the results are consistent with the UV-vis diffuse reflectance spectrum of Co/Co_*x*_O_*y*_@mC_600_.

#### Optimization of photocatalytic conditions for degrading MG

In addition, the optimal experimental conditions were investigated to achieve good MG degradation efficiency of Co/Co_*x*_O_*y*_@mC_600_. The usage of the catalyst and pH value of the solution were optimized. Fig. S9a† shows that the MG degradation rate by the catalyst increases with increase of dosage from 0.05 to 0.2 g L^−1^, approaching to 95% at 0.2 g L^−1^. Evidently, the *k* value of Co/Co_*x*_O_*y*_@mC_600_ with a dosage of 0.2 g L^−1^ is 1.83-fold that of Co/Co_*x*_O_*y*_@mC_600_ (0.05 g L^−1^, Fig. S9b†). Moreover, the pH effect of the polluted solution on the degradation ability was investigated, and the result are illustrated in Fig. S9c,† in which no distinct change is found among the MG degradation behaviors by Co/Co_*x*_O_*y*_@mC_600_. This finding indicates that the degradation kinetics against the catalyst is independent from the pH value of polluted water.

#### Cycling test and stability of the Co/Co_*x*_O_*y*_@mC_600_ catalyst

**Fig. 5:**
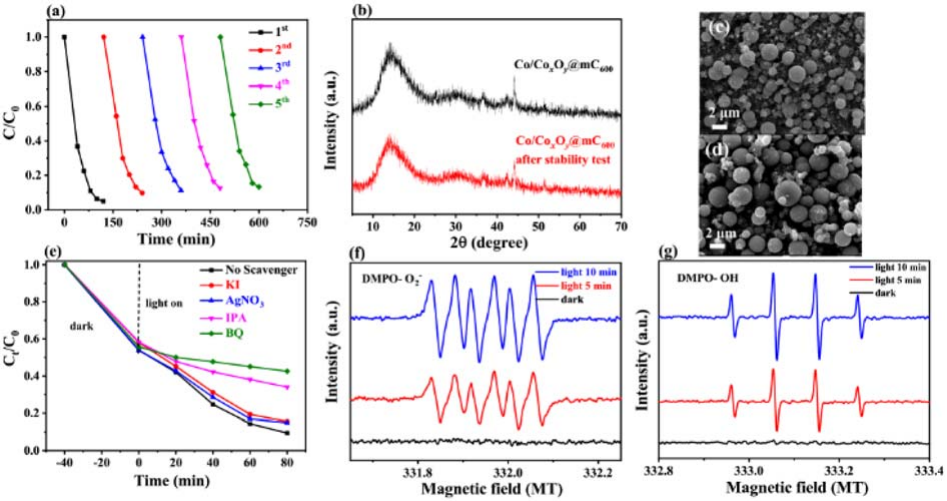
(a) Cyclic experiment of photocatalytic degradation of MG. (b) XRD pattern and (c and d) SEM images before and after five times of photodegradation test. (e) Degradation curves based on the different scavengers for MG photodegradation. ESR spectra of (f) DMPO-˙O_2_^−^, and (g) DMPO-˙OH for Co/Co_*x*_O_*y*_@mC_600_ in dark and under visible light irradiation for 5 min and 10 min.

The recyclability of Co/Co_*x*_O_*y*_@mC_600_ toward MG degradation was assessed. Fig. 5a shows that no substantial change in the photocatalytic activity is obtained after five cycles. After regeneration, the removal rates of MG by Co/Co_*x*_O_*y*_@mC_600_ are 95.0%, 90.3%, 88.8%, 87.5%, and 86.7%. Consequently, the good reusability of the Co/Co_*x*_O_*y*_@mC_600_ catalyst can be achieved.

The XRD pattern and SEM characterizations of the developed catalyst before and after used were employed to investigate the change in their features and the stability of basic performances of Co/Co_*x*_O_*y*_@mC_600_ (Fig. 5b–d). After five runs, no clear change in surface morphology and crystal structure of the catalyst are found, thereby indicating the excellent stability of Co/Co_*x*_O_*y*_@mC_600_.

### Degradation mechanism analysis

#### Investigation of active species and ESR testing

Active species trapping experiments were performed to deduce the migration path of the photogenerated electrons and holes in the developed photocatalysts. Fig. 5e displays the photocatalytic processes of the Co/Co_*x*_O_*y*_@mC_600_ catalyst in the presence of different quenchers, including KI, isopropyl alcohol (IPA), AgNO_3_, and *p*-benzoquinone (BQ), which are known as effective h^+^ trapping agent, ˙OH scavenger, e^−^ trapping agent, and ˙O_2_^−^ scavenger, respectively. Apparently, the photodegradation rates toward the photodegradation of MG decline in the following order: BQ (0.0032 min^−1^) < IPA (0.0065 min^−1^) < AgNO_3_ (0.0174 min^−1^) < KI (0.0176 min^−1^) (Fig. 5f and S10†). Accordingly, ˙O_2_^−^ and ˙OH are the most important reactive species, while e^−^ and h^+^ are the minor reactive species in the photocatalytic process.

Fig. 5g shows that no substantial peak is present under dark condition for ˙O_2_^−^ measurement. Meanwhile, four apparent peaks with the high intensity of ˙O_2_^−^ peaks can be found under visible light irradiation. Likewise, no clear peak of ˙OH under dark condition is observed, but four distinct peaks with the peak intensity of 1 : 2 : 2 : 1 of ˙OH is obtained under visible-light irradiation. Integration radical trapping experiments and ESR measurement, ˙O_2_^−^ and ˙OH species are considered as the main active species for the photodegradation of MG.

#### Degradation intermediates and pathways of MG using Co/Co_*x*_O_*y*_@mC_600_

**Scheme 1:**
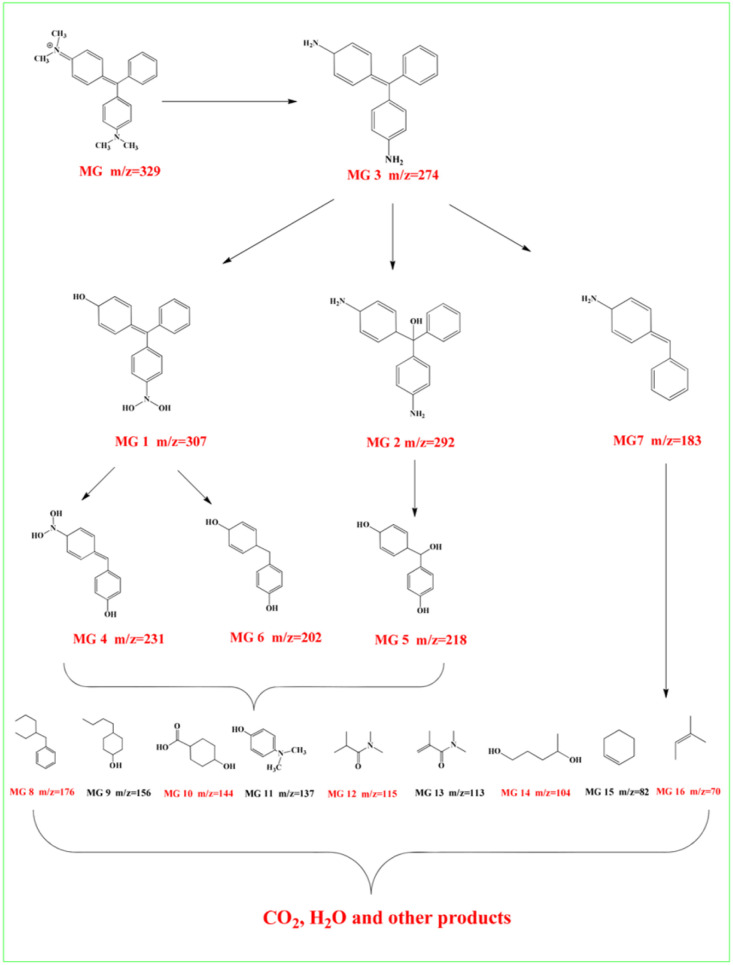
Possible pathway of the photodegradation of MG over Co/Co_*x*_O_*y*_@mC_600_.

The intermediates in the degradation process of MG using Co/Co_*x*_O_*y*_@mC_600_ were determined by using liquid organic acids, from which olefins and nitrogenous organic compounds were observed. In this regard, the degradation of MG could follow the possible pathway (Scheme 1). MG can be changed to MG 3 (*m*/*z* = 274) by losing methyl group, which can be further decomposed by three steps and characterized by high-performance liquid chromatography-mass spectrometry (HPLC-MS) (Fig. S11†). Firstly, MG 3 can be converted to MG 1 (*m*/*z* = 307) by hydroxyl substitution and deamination reaction,^60^ in which MG 1 forms MG 4 (*m*/*z* = 231), MG 5 (*m*/*z* = 218) and MG 6 (*m*/*z* = 202) through further ring opening reaction. Second, MG 3 is oxidized by ˙OH to generate MG 2 (*m*/*z* = 352), followed by breaking the carbon chain and hydroxyl substitution to produce MG 5 (*m*/*z* = 218).^61^

Third, MG 3 undergoes ring opening and deamination reaction, forming MG 7 (*m*/*z* = 183). These intermediates, that is, MG 4–7, are then decomposed to small molecular substances such as organic acids, phenols, olefins and nitrogen-containing organics, that is, MG 8–16, through further ring-opening reaction and methyl substitution reaction^62^ (Table S5†). Finally, CO_2_, H_2_O and other products are obtained through further oxidative decomposition.

#### Provided degradation mechanism for the degradation of MG

**Scheme 2:**
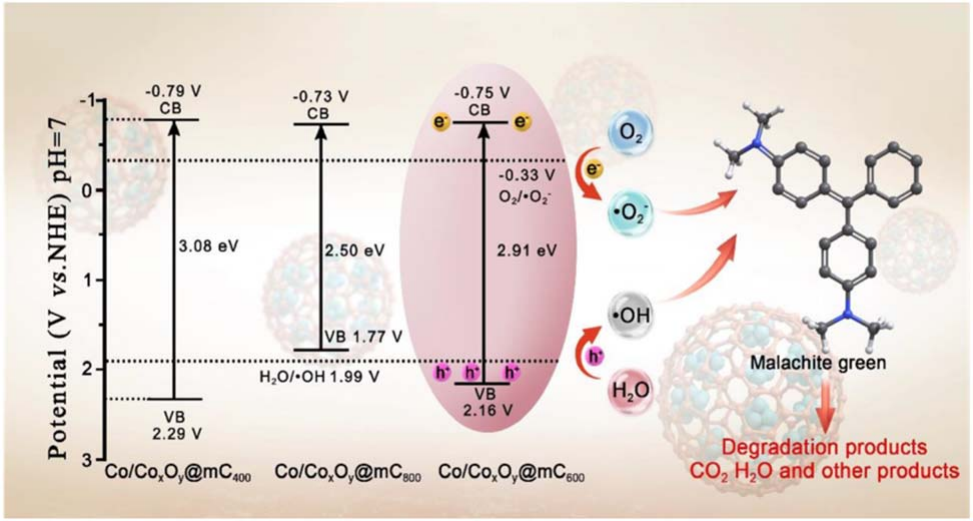
Schematic diagram for the possible charge separation and transfer in Co/Co_*x*_O_*y*_@mC_600_ under visible-light irradiation.

On the basis of the abovementioned analysis, the possible degradation mechanism of MG is proposed. As illustrated in Scheme 2, Co/Co_*x*_O_*y*_@mC_600_ can be excited with visible light illumination (eqn (7)) because of its narrow band gap of 2.91 eV, generating electron–hole pairs. Subsequently, photogenerated electrons can rapidly migrate to porous carbon layer, greatly improving the separation efficiency of photogenerated charge carriers. Given that the CB of Co/Co_*x*_O_*y*_@mC_600_ located at −0.75 V (*vs.* NHE) is more negative than *E*^0^ (O_2_/˙O_2_^−^) (−0.33 V *vs.* NHE),^63^ the dissolved O_2_ in solution and O_2_ on the Co/Co_*x*_O_*y*_@mC_600_ catalyst surface can be reduced by photogenerated electrons to yield ˙O_2_^−^ (eqn (8)). Moreover, considering that the VB of Co/Co_*x*_O_*y*_@mC_600_ (2.16 V *vs.* NHE) is more positive than H_2_O/˙OH (1.99 V *vs.* NHE), H_2_O could react with holes, thereby producing ˙OH^64^ (eqn (9)). These active species, that is, ˙O_2_^−^, ˙OH, and h^+^, can react with MG molecules, thereby degrading MG (eqn (10)). The reaction processes can be illustrated as follows:

7 Co/Co_*x*_O_*y*_@mC_600_ + *hν* → h^+^ + e^−^,

8 O_2_ + e^−^ → ˙O_2_^−^,

9 OH^−^ + h^+^ → ˙OH,

10 MG + active species (˙O_2_^−^/˙OH/h^+^) → CO_2_ + H_2_O + other products,

### Biotoxicity of MG degraded product tests

Antibacterial experiments were performed in *E. coli* suspension by culturing with aqueous solution containing MG after treatment with Co/Co_*x*_O_*y*_@mC_600_ to verify the biotoxicity of DPs of MG. Fig. S12a† shows the antibacterial state of blank control and solution containing DPs at different periods during photocatalytic. A small even negative optical density at 600 nm (OD_600_) indicates that the biological toxicity of the pure solution is high. However, after illumination for 60 min, the OD_600_ value increases to 0.537, demonstrating the low antibacterial ability of DPs. During degradation, the OD_600_ value of the treated solution increases to 0.826 after the light irradiation for 80 min.

This finding indicates that the toxicity of MG residues decreases, showing low antibacterial activity. Meanwhile, the experimental result of bacteriostatic circle was also evaluated (Fig. S12b†). After treatment with pure MG solution, the inhibition zone is 19 mm, indicating its good antibacterial activity. After photodegradation under light irradiation for 80 min, the inhibition zone of the solution containing DPs decreases to 10 mmm, indicating the declined antibacterial effect of DPs.

## Experimental

Some additional experimental descriptions such as materials and reagents, preparation of poly(2-methoxy-5-heptyoxyl terephthalic acid) (pbdc), basic characterizations, test for radical trapping, and electrochemical tests are supplied in the S1 section (see the ESI†).

### Preparation of polyMOF(Co)

PolyMOF(Co) was prepared using the modified method according to our previous work.^65^ Briefly, the as-synthesized pbdc (0.085 mmol, 28.73 mg) was dissolved in 30 mL of the mixed solution containing DMF and ethanol (*V*_DMF_ : *V*_ethanol_ = 1 : 1). Afterward, Co(NO_3_)_2_·6H_2_O (0.0429 mmol, 50 mg) was dissolved in the above solution. The mixture was transferred into a Teflon-linked stainless-steel autoclave and kept at 80 °C for 60 h. Then, the white solid powder was obtained by washing with DMF and ethanol. Finally, polyMOF(Co) was obtained after dried in vacuum at 60 °C.

### Preparation of the series of Co/Co_*x*_O_*y*_@mC hybrids

Co/Co_*x*_O_*y*_@mC was obtained by carbonizing polyMOF(Co) at various temperature. Briefly, the prepared polyMOF(Co) was loaded in a porcelain boat, which was located in the middle of the quartz tubular. Subsequently, the pyrolysis reaction was conducted at different temperature (400 °C, 600 °C, and 800 °C) and kept for 2 h under N_2_ atmosphere. After the temperature cooled down to 25 °C, the black powders were collected, which were defined as Co/Co_*x*_O_*y*_@mC_*t*_ (*t* = 400, 600, 800, representing different pyrolysis temperature).

### Adsorption ability of the series of Co/Co_*x*_O_*y*_@mC hybrids toward MG

The adsorption performance of Co/Co_*x*_O_*y*_@mC hybrid toward MG was evaluated at each adsorption process. Generally, Co/Co_*x*_O_*y*_@mC hybrid (10 mg) was separately dispersed in 50 mL of the MG solution with different concentrations by using ultrasonic treatment. Afterward, the dispersion was stirred at 400 rpm by magnetic stirring at 25 °C for 1 h. Co/Co_*x*_O_*y*_@mC was removed from the solution by centrifugation. The absorbance of MG residue was determined at 426 nm using a UV-vis spectrophotometer. The adsorption capacity of Co/Co_*x*_O_*y*_@mC for MG was estimated according to the eqn (11):

11 *q*_e_ = (*C*_0_ − C_*t*_)*V*/*m*,

where *C*_0_, *C*_*t*_, *V* (mL), and *m* (g) are the initial concentration of MG, the concentration of MG at time *t* (min), the volume of solution, and the mass of the series of Co/Co_*x*_O_*y*_@mC hybrids, respectively.

### Photocatalytic measurements of the series of Co/Co_*x*_O_*y*_@mC hybrids toward MG

The photodegradation rates of the series of Co/Co_*x*_O_*y*_@mC hybrids toward MG were evaluated at room temperature. For example, Co/Co_*x*_O_*y*_@mC_600_ (5 mg) was decentralized in the MG solution (50 mL, 80 mg L^−1^), following by ultrasonic treatment for 30 min. The pH of acid or alkali of the treated water systems was modulated using NaOH or HCl. Subsequently, the prepared mixture was agitated for 40 min in dark at room temperature to obtain the equilibrium of the adsorption–desorption. Then, a 300 W Xenon arc lamp with a 420 nm filter was used to irradiate the mixture within the pre-determined period. Afterward, 3 mL of the supernatant was collected and centrifuged to separate the used Co/Co_*x*_O_*y*_@mC. The UV absorbance of the MG solution was measured at a wavelength of 426 nm. Moreover, the degradation rate of MG was determined by recording the UV absorbance of the supernatant, which is calculated by the eqn (12):

12 *ϕ* = (*C*_0_ − *C*_*t*_)/*C*_0_,

where *C*_0_ and *C*_*t*_ are the MG concentrations at the beginning of light irradiation and time *t*, respectively. Further, the parameters for photodegradation, including the catalyst dosage (0.1, 0.2, and 0.3 g L^−1^), and pH value (pH = 4, 7, and 10) of the MG solutions were studied to determine the optimal photodegradation efficiency. In addition, the cyclic runs of MG degradation were performed to evaluate the stability of Co/Co_*x*_O_*y*_@mC. During measurement, the used Co/Co_*x*_O_*y*_@mC catalyst was gathered by centrifugation after each cycle, and washed with ethanol several times for the next cycle. The whole procedure for the degradation of MG and refreshment were repeated for five runs. Furthermore, the surface morphologies and crystal structures of the used catalysts were conducted to evaluate the stability of the basic performances.

### Biological toxicity evaluation of degraded products

*Escherichia coli* (*E. coli* ATCC25922) was used as a model for analysing the effect of degraded products (DPs) of MG antibacterial ability. The agar diffusion tests were employed to evaluate the biological toxicity of DPs, which was obtained by centrifugation for removing the catalyst after degradation tests. Firstly, the diluted *E. coli* suspension was prepared by adding 100 μL of *E. coli* (10^8^ CFU mL^−1^) into 900 μL of culture medium. Following, the samples (*ϕ* = 6 mm) were cut manually in the agar plate, then incubated with *E. coli* suspension (10^7^ CFU mL^−1^), 100 μL of MG solution, and 100 μL of DPs solution. After incubating at 37 °C for 24 h, the diameter of inhibition zone was measured. Further, six experimental groups were conducted for evaluating the antibacterial effect of MG residuals after different degradation time, including the pure MG solution, the MG solution after treating with the catalyst for 20 min, and the mixtures of DPs solutions with *E. coli* suspension after 20, 40, 60, and 80 min. The blank control group was prepared by mixing 50 μL of sterilized saline solution (0.9% NaCl) and 950 μL of *E. coli* suspension (10^7^ CFU mL^−1^). All experimental and controlled groups were incubated for 24 h at 37 °C. Finally, UV-vis spectra were employed to evaluate the optical density (OD) values of the bacterial culture at 600 nm.

## Conclusions

The polyMOF(Co) microspheres were synthesized using a polyether ligand as building block, and were calcined to construct a series of Co/Co_*x*_O_*y*_@mC photocatalysts at high temperature under N_2_ atmosphere for the photodegradation of MG under visible-light illumination. Given the carbon-rich functionality and homogeneously distributed cobalt sites, multiple active sites such as metallic Co, CoO, and Co_3_O_4_ nanoparticles were yielded, which were embedded within mesoporous carbon spheres. By contrast, Co/Co_*x*_O_*y*_@mC_600_ not only exhibited a large specific surface area (236 m^2^ g^−1^) and evenly distributed metal particles, but also demonstrated superior photo-electron transfer efficiency and enhanced the separation ability of electron–hole pairs. This result indicated the great absorption performance (314 mg g^−1^ in 120 min) and degradation ability (95% within 80 min under visible light) of MG to the other catalysts, with good regeneration. ESR analysis illustrated that ˙O_2_^−^ and ˙OH were the main active substances in photocatalytic degradation. In addition, the antibacterial result indicated that the biological toxicity of degradation products of MG treated with Co/Co_*x*_O_*y*_@mC_600_ can be ignored. This work provided a robust strategy for the development of efficient photocatalysts based on polyMOFs for the removal and degradation of organic dyes, thereby showing great potential in environmental remediation.

## Author contributions

Shuai Zhang: formal analysis, writing-review & editing. Hao Dang and Feilong Rong: formal analysis, investigation. Shunjiang Huang: formal analysis, investigation, writing-review & editing. Minghua Wang: formal analysis, methodology. Lijun Hu: data curation, conceptualization. Zhihong Zhang: writing-review & editing, supervision.

## Conflicts of interest

There are no conflicts to declare.

## Supplementary Material

RA-012-D2RA04906F-s001
