# Supplementary material for: Multiple cobalt active sites evenly embedded in mesoporous carbon nanospheres derived from a polymer-metal-organic framework: efficient removal and photodegradation of malachite green
Source: RSC Adv. 2022 Nov 10;12(50):32307–17. doi: 10.1039/d2ra04906f (PMC9648500; doi:10.1039/d2ra04906f)
Supplement: RA-012-D2RA04906F-s001 [file RA-012-D2RA04906F-s001.pdf]

Electronic Supplementary Material (ESI) for RSC Advances.
This journal is © The Royal Society of Chemistry 2022

Supplementary Material for

Multiple cobalt active sites evenly embedded within mesoporous carbon nanospheres derived from polymer-metal-organic framework: Efficient removal and photodegradation of malachite green

Shuai Zhang, Hao Dang, Feilong Rong, Shunjiang Huang, Minghua Wang, Lijun Hu, Zhihong Zhang*

College of Material and Chemical Engineering, Zhengzhou University of Light Industry, Zhengzhou 450001, China

* Corresponding authors. E-mail addresses: 2006025@zzuli.edu.cn (Z. Zhang).

Content

S1. Experimental section

S1.1 Materials and reagents

S1.2 Preparation of poly-terephthalic acid (pbdc-8)

S1.3 Basic characterizations

S1.4 Test for radical trapping

S1.5 Electrochemical tests

S2. Basic characterizations of polyMOF(Co)

S3. Basic characterizations of the series of Co/Co_xO_y@mC hybrids

S4 Photocatalytic degradation of malachite green

S1. Experimental section

S1.1 Materials and reagents

2,5-dihydroxyterephthalic acid, 1,8-dibromooctane, cobaltous nitrate hexahydrate, *N,N*-dimethylformamide (DMF), tetrahydrofuran and all other chemicals were purchased from Aladdin Reagent Co. Ltd. All reagents were analytical grade reagents and used without further purification. The deionized water used throughout all experiments.

S1.2 Preparation of poly-terephthalic acid (pbdc)

The ligand of pbdc was synthesized according to our previous work [1].

S1.3 Basic characterizations

The crystal structure of the precursor and final product were both analyzed by TongdaTD-3500X-ray powder diffractometer with Cu-Ka radiation. Raman spectra were gained from a Renishaw inVia Raman spectrometer with a solid-state laser (excitation at 532 nm) at room temperature in the range of 100-3000 cm^{-1}. The chemical structure was probed through by the Fourier transform infrared spectroscopy (FT-IR) (Bruker TENSOR27, Germany). Surface electronic structure was analyzed by X-ray photoelectron spectroscopy (XPS) by using a VG ESCALAB HP photoelectron spectrometer equipped with an analyzer and preparation chambers. Inductively coupled plasma mass spectrometry (ICP-MS, PerkinElmer, Elan9000) was used to determine the Co content of Co/Co_xO_y@mC hybrids. The surface morphology of the synthesized catalysts was investigated through scanning electron microscope (SEM, JSM-6490LV, Japan) and transmission electron microscopy (TEM, Hitachi H-800, Tokyo, Japan).

Also, the specific surface areas of all samples were calculated by the Brunauer-Emmett-Teller (BET) method.

S1.4 Test for radical trapping

The experiments for radical trapping were performed using the same process as the measurements of photocatalytic activity except for addition of different scavengers, including KI (2 mM), benzoquinone (BQ) (2 mM), $AgNO_3$ (2 mM), and isopropyl alcohol (IPA) (2 mM), which were employed as scavengers to trap the catalytic active sites hole (h^+), superoxide radicals ($^\bullet O_2^-$), electron (e^-), and hydroxyl radicals ($^\bullet OH$), respectively. Moreover, electron spin resonance (ESR) spectrometry was applied to determine the types of radical species on a JES-FA spectrometer under visible light irradiation by using 5,5-dimethyl-l-pyrroline N-oxide (DMPO) as spin trapping agents.

S1.5 Electrochemical analysis

The photocurrent, Mott-Schottky plots, and electrochemical impedance spectroscopy (EIS) were measured on CHI660D electrochemical analyzer in the three-electrode system with a 300 W Xe arc lamp as light source. The three-electrode system takes a platinum wire as counter electrode, FTO (1.5 × 3.0 cm) coated with photocatalyst as working electrode and an Ag/AgCl (saturated KCl) as reference electrode. Typically, the as-prepared photocatalyst (8.0 mg) was dispersed in absolute ethanol (1.0 mL) with an ultrasonic bath for 30 min. After that, the photocatalyst (50 μL) was coated onto the FTO and dried naturally. The photocurrent and Mott-Schottky plots were recorded in 0.5 M Na_2SO_4 solution. Meanwhile, the EIS was performed in a 0.1 M KCl solution containing 5 mM $[Fe(CN)_6]^{3-/4-}$.

S2. Basic characterizations of polyMOF(Co)

Figs. S1a indicates that polyMOF(Co) exhibits sphere-like shape, which comprising large amounts of micro- and nano-spheres with the different diameters (0.5-2μm) and smooth surface. Occasionally, two or several spheres are grown together and formed irregular shape (**Fig. S1b**), further confirming by the TEM image (**Fig. S1c**). The TEM image of polyMOF(Co) also displays the smooth surface, while the high-resolution TEM image (**Fig. S1e**) does show the blurry lattice fringe spacing of 0.204 nm that is ascribed to Co (111) [2]. In addition, the elemental mapping indicates that Co, C, O are distributed evenly. The EDX spectrum (**Fig. S2a**) shows that the atomic percentages of Co, O and C content are 15.25%, 39.48% and 46.27%, respectively. **Fig. S2b** indicates that the peaks are located at 7.62°, 9.72°, 13.44°, 19.40°, and 26.24° in the XRD pattern of polyMOF(Co), which is similar with Co-BDC MOF [3], indicating the successful preparation of polyMOF(Co). Additionally, it can be observed amorphous carbon peak located at 20.44°, which can be explained by the utilization of polymer ligand for the preparation of polyMOF. **Fig. S2c** indicates the FT-IR spectrum of polyMOF(Co), in which the absorption peaks at 2854 cm^{-1} and 2940 cm^{-1} due to $-CH_2-$ and $-CH_3$ are obtained for polyMOF(Co). These groups are apparently originated from polymer ligand.

Fig. S2d depicts the N_2 adsorption-desorption isotherm of polyMOF(Co) displays a H_3 hysteresis loop. As the result a specific surface area of 85.7 m^2g^{-1} and the pore diameters of 7.55 nm are observed (**Fig. S2d, inset**). As compared, the specific surface area of polyMOF(Co) is substantially smaller than those of common MOFs [4]. It is

mainly due to the usage of polymer ligand, and the pore was filled by the methylene spacers [5]. Meanwhile, the Raman spectrum (**Fig. S2e**) indicates that the peak located at 1613 cm^{-1} is ascribed to phenyl ring of the polymer ligand. All results indicate polyMOF(Co) was synthesized successfully.

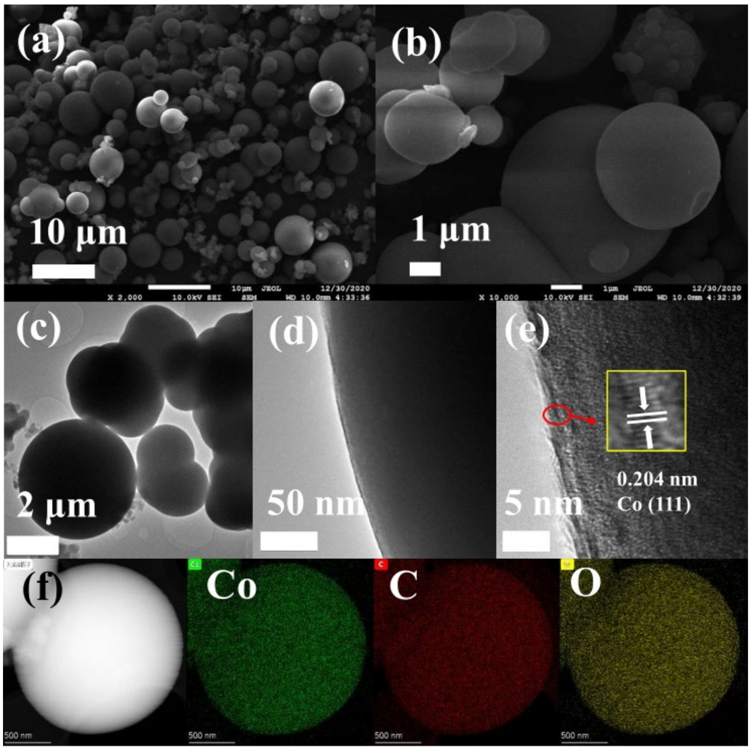

Fig. S1 (a, b) Low- and high-magnification SEM images, (c, d, e) low-, high-magnification, and high-resolution TEM images, and (f) EDX mapping images of polyMOF(Co).

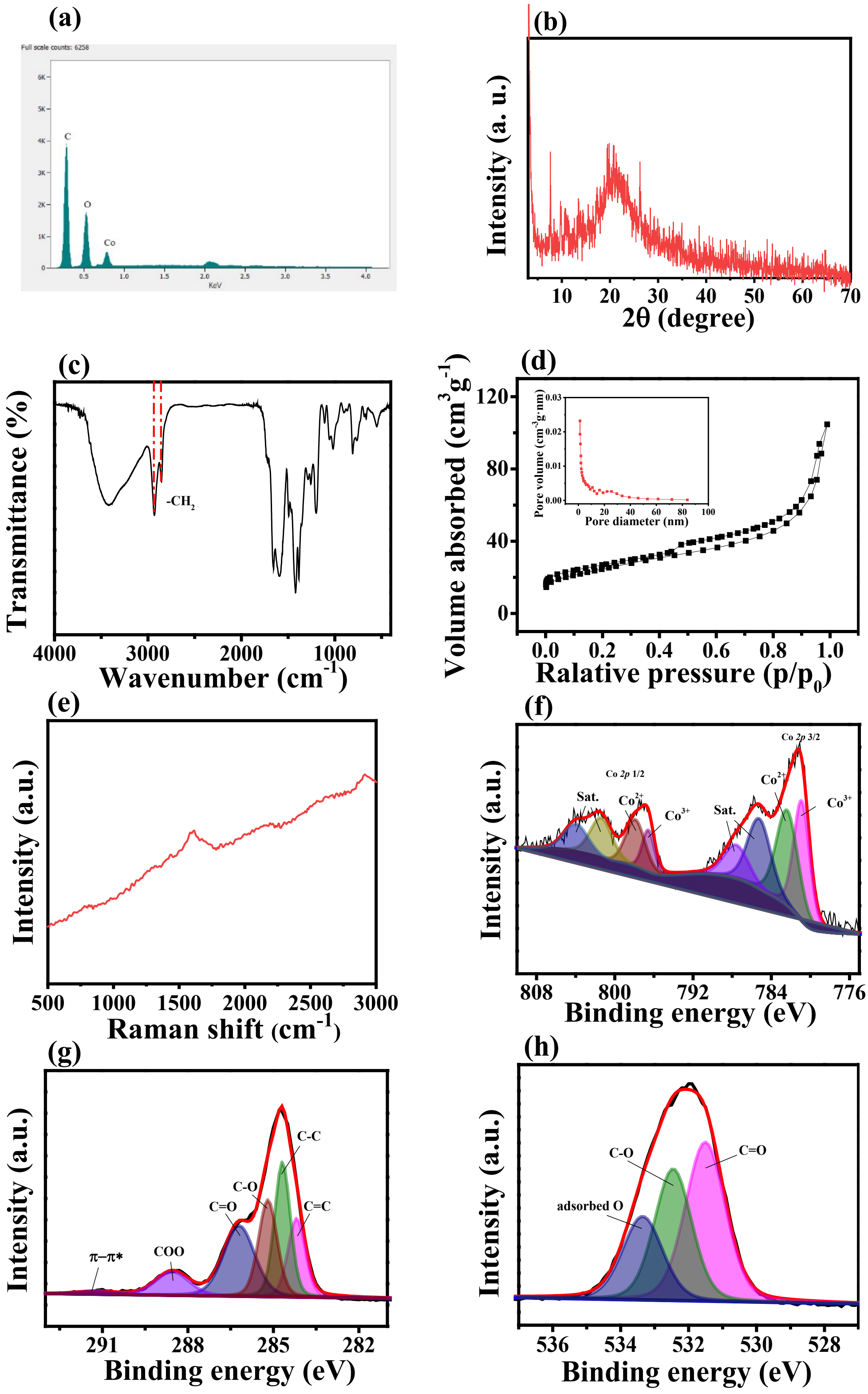

Fig. S2 (a) EDX spectrum, (b) XRD pattern, (c) FT-IR spectrum, (d) N_2 adsorption–desorption isotherm, inset image: corresponding pore size distribution curve and high-resolution, (e) Raman spectra, (f) Co 2*p*, (g) C 1*s*, and (h) O 1*s* XPS spectra of

polyMOF(Co).

S3. Basic characterizations of the series of Co/Co$_x$O$_y$@mC hybrids

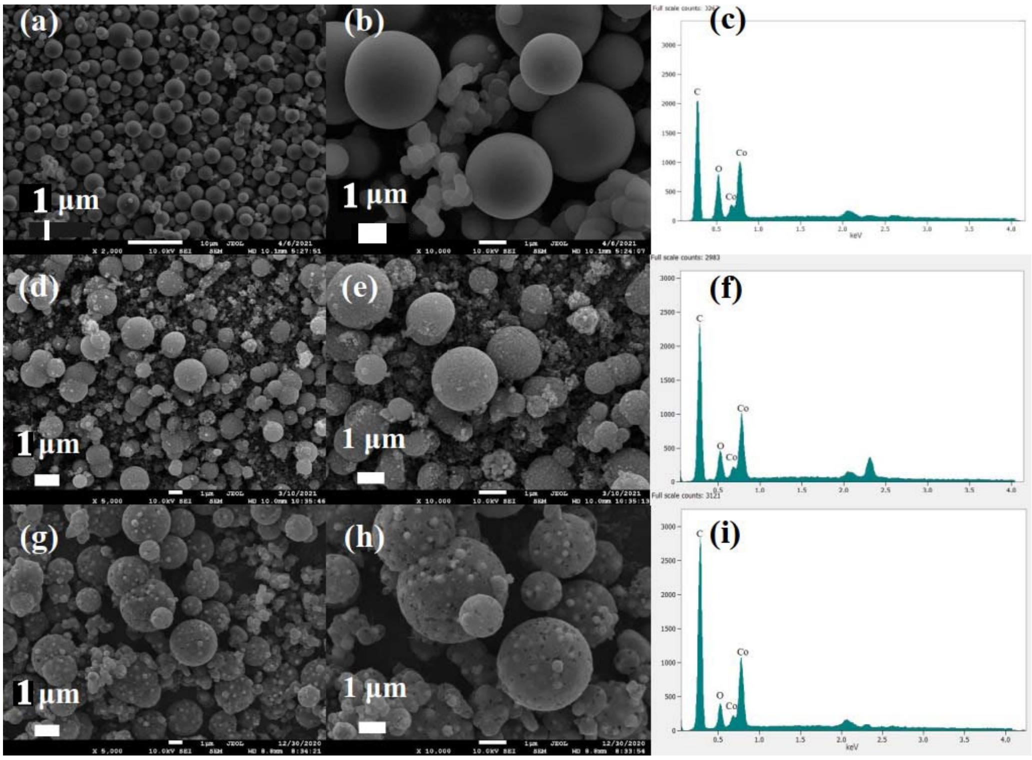

Fig. S3 Low- and high-magnification SEM images and EDX spectra of (a, b, c) Co/Co$_x$O$_y$@mC$_{400}$, (d, e, f) Co/Co$_x$O$_y$@mC$_{600}$, and (g, h, i) Co/Co$_x$O$_y$@mC$_{800}$ hybrids.

Table S1 The content of each component containing in all samples

**Sample**	**Atomic%**		**ICP-MS**
	**C 1*s***	**O 1s**	**Co (%)**
polyMOF(Co)	46.27±0.03	39.48±0.08	12.1±0.1
$Co/Co_xO_y@mC_{400}$	57.24±0.05	21.81±0.12	15.5±0.6
$Co/Co_xO_y@mC_{600}$	65.37±0.1	19.25±0.14	18.11±3.6
$Co/Co_xO_y@mC_{800}$	69.99±0.05	16.48±0.4	18.43±5.4

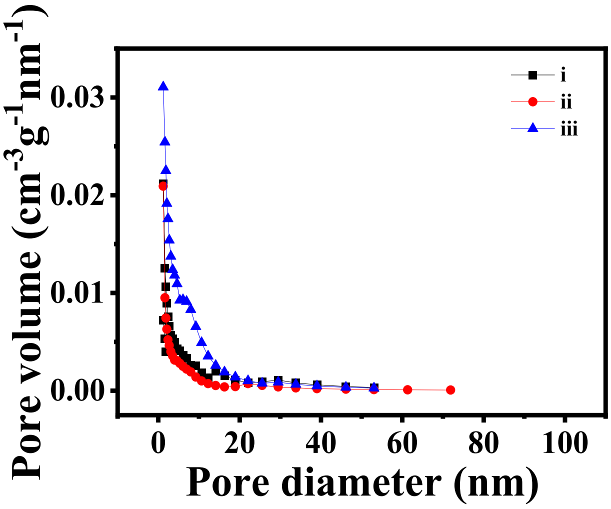

Fig. S4 The corresponding pore size distribution curves of (i) Co/Co_xO_y@mC_{400}, (ii) Co/Co_xO_y@mC_{600}, and (iii) Co/Co_xO_y@mC_{800}.

Table S2 BET surface areas, pore diameters, and pore volumes of the series of Co/Co_xO_y@mC hybrids.

**Sample**	**BET surface area (m^2 g^{-1})**	**Pore diameter (nm)**	**Pore volume (cm^3 g^{-1})**
polyMOF(Co)	85.7	7.55	0.1619
Co/Co_xO_y@mC_{400}	161	3.50	0.1415
Co/Co_xO_y@mC_{600}	236	2.37	0.1400
Co/Co_xO_y@mC_{800}	264	3.61	0.2382

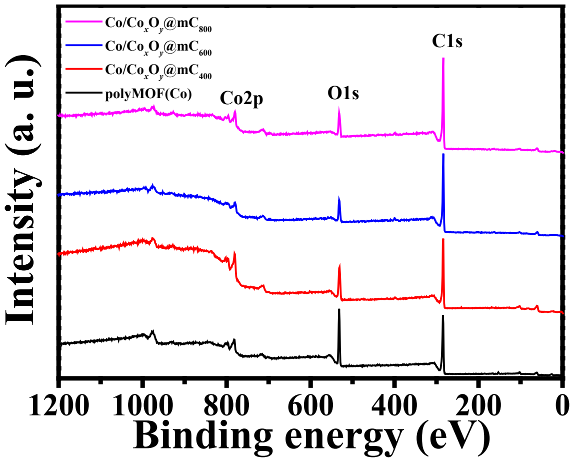

Fig. S5 XPS survey scan spectra of polyMOF(Co), Co/Co$_x$O$_y$@mC$_{400}$, Co/Co$_x$O$_y$@mC$_{600}$, and Co/Co$_x$O$_y$@mC$_{800}$.

S4 Photocatalytic degradation of malachite green

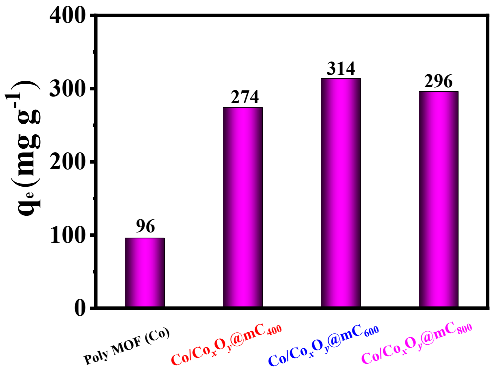

Fig. S6 Comparison of maximum adsorption capacity of malachite green on different adsorbents

Table S3 Kinetic parameters of kinetic fitting for the removal of MG using diverse catalysts of polyMOF(Co) and the series of Co/Co$_x$O$_y$@mC hybrids.

	Fitting parameters of quasi first order dynamics			Fitting parameters of quasi second order dynamics		
	K_1 min^{-1}	q_e mg·g^{-1}	R^2	K_2 g (min·mg)$^{-1}$	q_e mg·g^{-1}	R^2
polyMOF(Co)	0.0301	88	0.9803	0.0003540	116	0.9973
Co/Co$_x$O$_y$@mC$_{400}$	0.0247	302	0.9805	0.0000278	455	0.9797
Co/Co$_x$O$_y$@mC$_{600}$	0.0257	328	0.9641	0. 0000473	435	0.9893
Co/Co$_x$O$_y$@mC$_{800}$	0.0268	346	0.9631	0.0000246	500	0.9533

Table S4 Langmuir and Freundlich adsorption isotherm model parameters

Langmuir fitting parameters			Freundlich fitting parameters		
k_3	q_m (mg g^{-1})	R^2	k_4 (L mg^{-1})	$1/n$	R^2
0.4283	308	0.9875	128.58	0.2237	0.8970

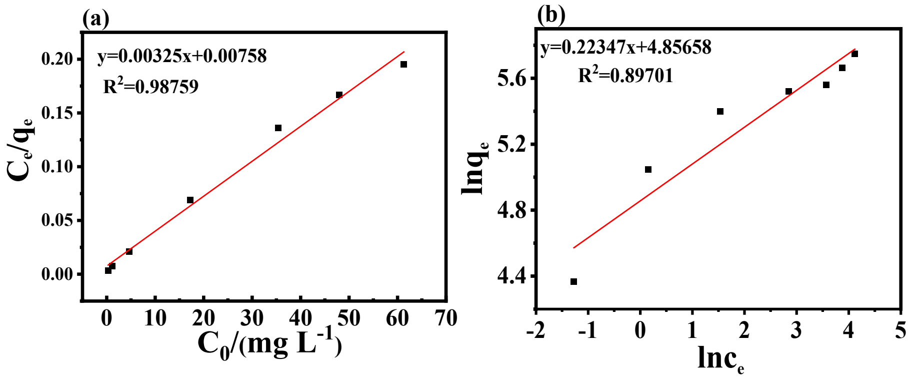

Fig. S7 (a) Adsorption equation fitting curve of Langmuir isotherms, and (b) adsorption equation fitting curve of Freundlich isotherms

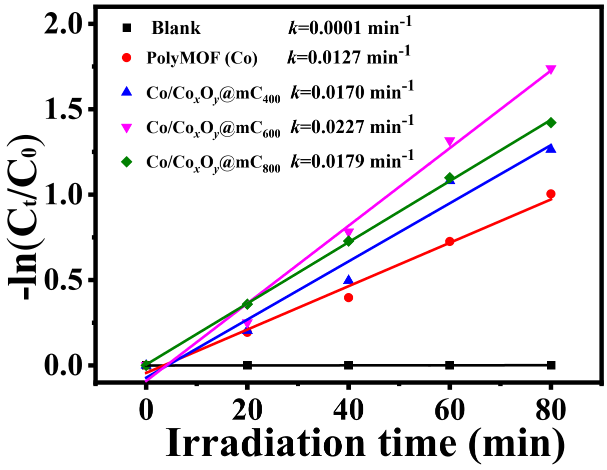

Fig. S8 The pseudo-first-order kinetic fitting curves of polyMOF(Co), $Co/Co_xO_y@mC_{400}$, $Co/Co_xO_y@mC_{600}$, and $Co/Co_xO_y@mC_{800}$ for photocatalytic degradation of MG

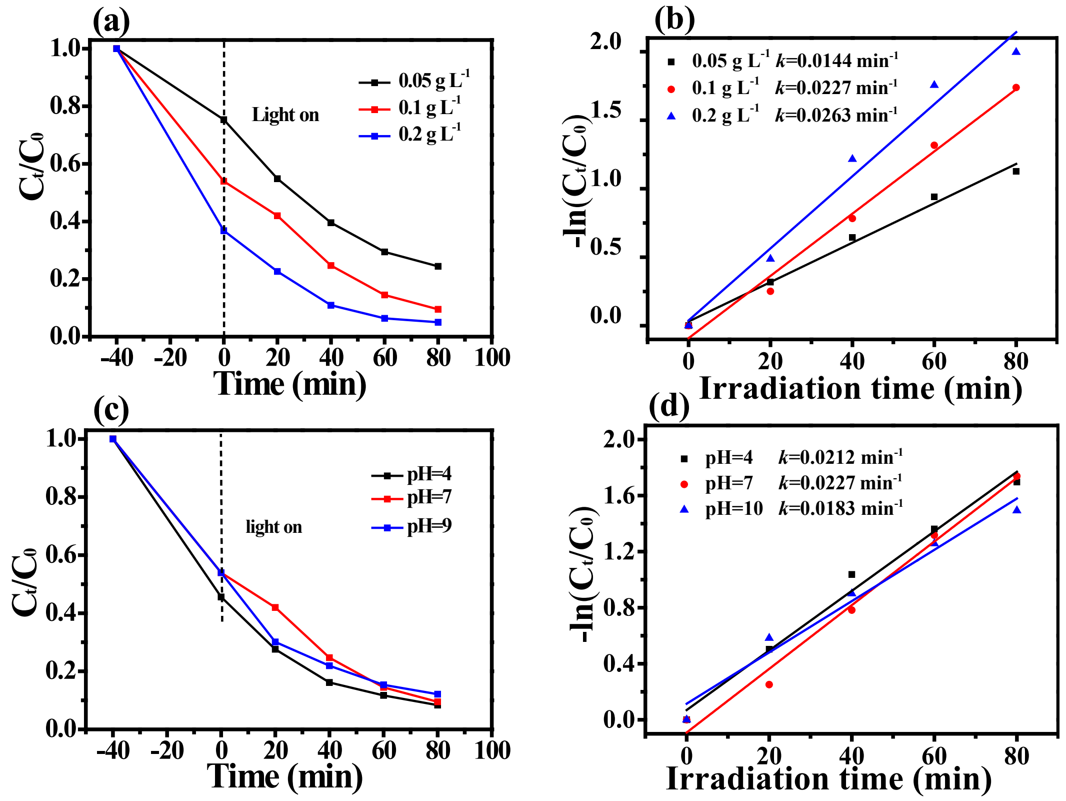

Fig. S9 The effects of (a) the amount of catalyst, (c) pH values, on the MG degradation and (b, d) their corresponding reaction rate constants. Reaction parameters: (a) [MG] = 80 mg L^{-1}, [pH] = 7.0, and [T] = 25 °C; (c) [MG] = 80 mg L^{-1} and [Co/Co_xO_y@mC $_{600}$] = 0.1 g L^{-1}; [T] = 25 °C.

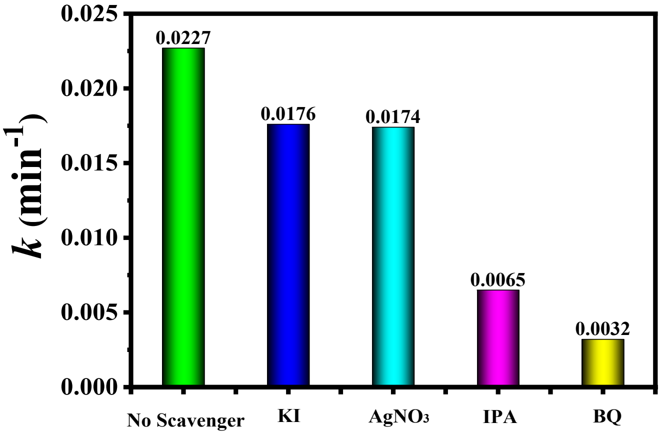

Fig. S10 The reaction rate constants *k* based on the different scavengers for MG photodegradation

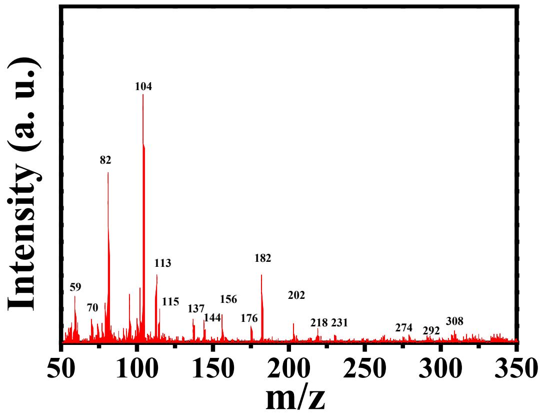

Fig. S11 MS spectra of the MG solution eluted after reaction time of 80 min over $Co/Co_xO_y@mC_{600}$.

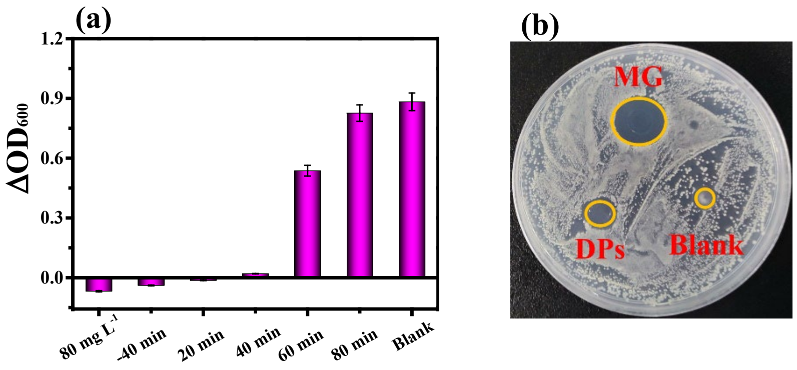

Fig. S12 (a) The OD_{600} values of *E. coli* in blank control and degraded solution groups using $Co/Co_xO_y@mC_{600}$ and (b) the inhibition zone of blank, pure MG solution, and solution containing DPs toward the *E. coli* growth.

Table S5 Intermediate products of MG during the treatment process detected by LC-MS/MS.

Compounds	m/z	Molecular formula	Possible molecular structure
**MG**	**329**	**$C_{23}H_{25}N_2$**	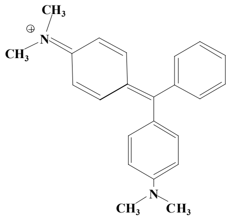
**MG 1**	**307**	**$C_{19}H_{17}NO_3$**	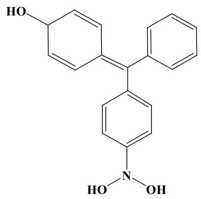
**MG 2**	**292**	**$C_{19}H_{20}N_2O$**	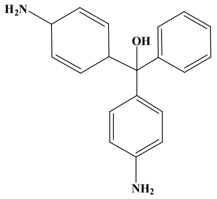
**MG 3**	**274**	**$C_{19}H_{18}N_2$**	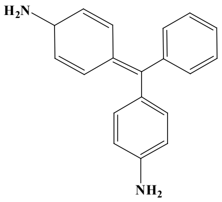
**MG 4**	**231**	**$C_{13}H_{13}NO_3$**	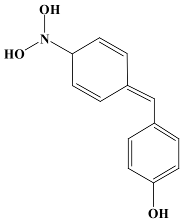
**MG 5**	**218**	**$C_{13}H_{14}O_3$**	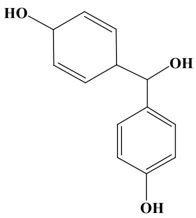
**MG 6**	**202**	**$C_{13}H_{14}O_2$**	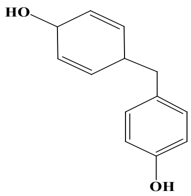

**MG 7**	**183**	**$C_{13}H_{13}N$**	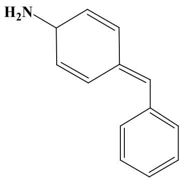
**MG 8**	**176**	**$C_{13}H_{20}$**	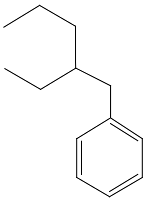
**MG 9**	**156**	**$C_{10}H_{20}O$**	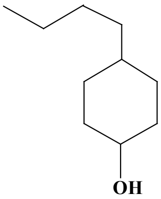
**MG 10**	**144**	**$C_7H_{12}O_2$**	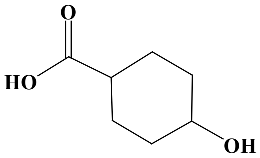
**MG 11**	**137**	**$C_8H_{11}NO$**	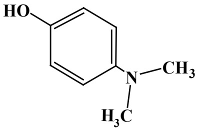
**MG 12**	**115**	**$C_6H_{11}NO$**	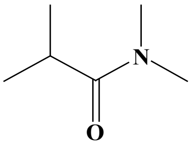
**MG 13**	**113**	**$C_6H_{13}NO$**	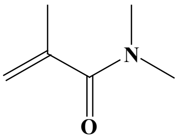
**MG 14**	**104**	**$C_5H_{12}O_2$**	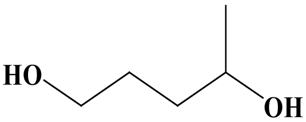
**MG 15**	**82**	**C_6H_{10}**	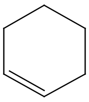
**MG 16**	**70**	**C_5H_{10}**	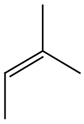

References:

1. Q. Jia, Y. Lou, F. Rong, S. Zhang, M. Wang, L. He, Z. Zhang and M. Du, *J. Mater. Chem. C*, 2021, **9**, 14190-14200.
2. H. Lv, X. Liang, G. Ji, H. Zhang and Y. Du, *ACS Appl. Mater. Interfaces*, 2015, **7**, 9776-9783.
3. Q. Zha, F. Yuan, G. Qin and Y. Ni, *Inorg. Chem.*, 2020, **59**, 1295-1305.
4. S. Ayala, Z. Zhang and S. M. Cohen, *Chem. Commun.*, 2017, **53**, 3058-3061.
5. H. Wu, Y. S. Chua, V. Krungleviciute, M. Tyagi, P. Chen, T. Yildirim and W. Zhou, *J. Am. Chem. Soc.*, 2013, **135**, 10525-10532.
